# Proteomic and Physiological Responses of *Kineococcus radiotolerans* to Copper

**DOI:** 10.1371/journal.pone.0012427

**Published:** 2010-08-26

**Authors:** Christopher E. Bagwell, Kim K. Hixson, Charles E. Milliken, Daniel Lopez-Ferrer, Karl K. Weitz

**Affiliations:** 1 Department of Environmental Sciences and Biotechnology, Savannah River National Laboratory, Aiken, South Carolina, United States of America; 2 Environmental Molecular Sciences Laboratory, Richland, Washington, United States of America; 3 Biological Sciences Division, Pacific Northwest National Laboratory, Richland, Washington, United States of America; University of Wisconsin-Milwaukee, United States of America

## Abstract

Copper is a highly reactive, toxic metal; consequently, transport of this metal within the cell is tightly regulated. Intriguingly, the actinobacterium *Kineococcus radiotolerans* has been shown to not only accumulate soluble copper to high levels within the cytoplasm, but the phenotype also correlated with enhanced cell growth during chronic exposure to ionizing radiation. This study offers a first glimpse into the physiological and proteomic responses of *K. radiotolerans* to copper at increasing concentration and distinct growth phases. Aerobic growth rates and biomass yields were similar over a range of Cu(II) concentrations (0–1.5 mM) in complex medium. Copper uptake coincided with active cell growth and intracellular accumulation was positively correlated with Cu(II) concentration in the growth medium (R^2^ = 0.7). Approximately 40% of protein coding ORFs on the *K. radiotolerans* genome were differentially expressed in response to the copper treatments imposed. Copper accumulation coincided with increased abundance of proteins involved in oxidative stress and defense, DNA stabilization and repair, and protein turnover. Interestingly, the specific activity of superoxide dismutase was repressed by low to moderate concentrations of copper during exponential growth, and activity was unresponsive to perturbation with paraquot. The biochemical response pathways invoked by sub-lethal copper concentrations are exceptionally complex; though integral cellular functions are preserved, in part, through the coordination of defense enzymes, chaperones, antioxidants and protective osmolytes that likely help maintain cellular redox. This study extends our understanding of the ecology and physiology of this unique actinobacterium that could potentially inspire new biotechnologies in metal recovery and sequestration, and environmental restoration.

## Introduction

*Kineococcus radiotolerans* was isolated within a shielded cell work area containing highly radioactive nuclear waste at the U.S. Department of Energy's Savannah River Site in Aiken, SC, USA [1]. *K. radiotolerans* is an orange pigmented, aerobic, zoosporic actinomycete belonging to the *Kineosporiaceae* family. Relatively little is known about the eco-physiology of these organisms despite their ubiquity, and often numerical dominance, in terrestrial [2], [3], arid [4], [5], [6], and aquatic environments [7], [8], [9]. *K. radiotolerans* is resistant to γ-irradiation, prolonged desiccation, and strong oxidants [1], [10]; attributes that are consistent with the known ecological distributions of these actinobacteria. It is apparent that *K. radiotolerans,* and related taxa [1], [11], [12], are well adapted to life in harsh environmental conditions, though the specific cellular and biochemical mechanisms that promote survival remain unexplored.

*K. radiotolerans* was recently shown to specifically and actively accumulate soluble copper within the cytoplasm and more intriguingly, colony formation was stimulated on solid plating medium supplemented with copper (100 µM final concentration) during chronic irradiation [10]. Copper is a critical cofactor for a number of metalloenzymes involved in energy metabolism and oxidative defense, but only trace levels are required. In excess, copper is extremely toxic; thus, cells exert strict homeostatic control over membrane transport and intracellular trafficking [13], [14]. Because of its reactivity as a redox active metal capable of participating in Fenton/Haber-Weiss reactions and the inherent toxicity of the metal; free, unbound copper is virtually absent within the cytoplasm of unstressed cells [14]. The intracellular fate of copper within *K. radiotolerans* has not been determined.

Copper metabolism has been studied extensively in a limited number of taxa, however disparate bacterial phyla often possess a resistance operon analogous to CopABYZ in *Enterococcus hirae* [15], [16] which relies upon a CPx-type ATPase (i.e., CopB) for the export of excess copper [2], [17]. *E. hirae* can tolerate copper concentrations as high as 5 mM [18], though at concentrations as low as 0.5 mM steady-state levels of the metallochaperone CopZ are subjected to proteolytic degradation, thus minimizing intracellular metal accumulation and undue oxidative stress [19]. Other model systems for copper resistance and homeostasis pertain to the Gram negative bacteria where heavy metal ions are either sorbed onto the outer membrane or localized within the periplasmic space [17], [20], [21]; consequently, physical sequestration and compartmentalization prevents interruption of intracellular processes [22]. The centerpiece to all resistance mechanisms studied to date is the strict regulation of intracellular copper flux. Because relatively little is known about metal accumulation and resistance pathways in Gram positive actinobacteria, cytoplasmic copper accumulation in *K. radiotolerans* is intriguing, especially the biochemical functionality permitting copper enhanced growth during γ-irradiation [10]. Intracellular copper accumulation is expected to require a metabolite(s) to sequester the heavy metal, as well as molecular mechanisms to mitigate cellular toxicity and production of reactive oxygen species via redox cycling. The goals of this study were to characterize growth coordinated physiological activities paired with global proteomic responses of *K. radiotolerans* across a range of copper concentrations to gain insight into metal homeostasis and cellular defense pathways.

## Results and Discussion

**Figure 1:**
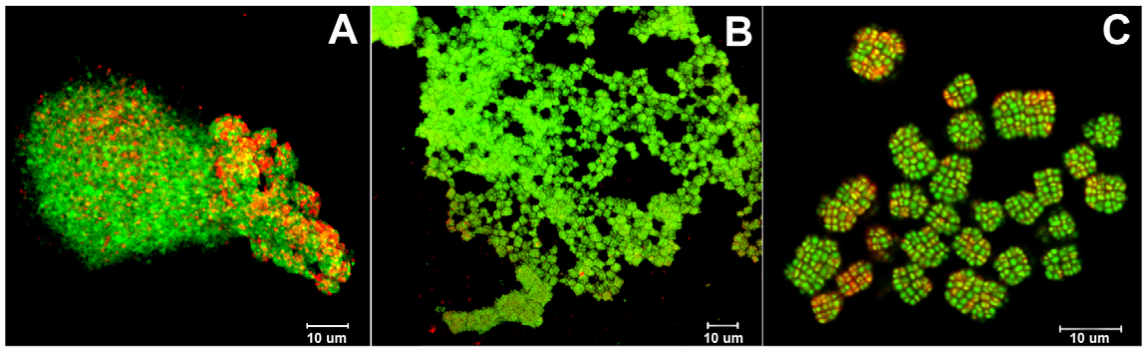
Morphological transformations of *K. radiotolerans* observed at mid-exponential (A.), late exponential (B.), and early stationary (C.) growth phases. Cells were collected from liquid culture (TGY), stained with acridine orange and viewed by confocal laser scanning microscopy.

The high GC content Gram positive actinobacteria are ubiquitous in the environment. They partake in intimate plant associations [9], [23], predominate in arid environments [4], [6], [24], and contribute to the weathering of rock [25], [26], paintings and monuments [27], [28], [29]. Numerous studies have also demonstrated routine detection and recovery from heavy metal enriched habitats [30]–[36], though relatively little is known about metal resistance and homeostasis pathways among the actinobacteria. Detailed physiological characterization of the actinobacteria is complicated, in part, by the complex morphogenesis exhibited by many species [37]–[39], prolonged dormancy or periods of inactivity [40], [41], and, as is the case for *K. radiotolerans,* extensive cell clumping in artificial growth media (Figure 1). Attempts to establish defined growth conditions by altering medium composition, oxygen tension, inclusion of reducing agents, and various combinations of amino acids, vitamins, growth factors, and detergents have so far failed to support growth of *K. radiotolerans*. It is probable that within a large clump (Figure 1A); individual cells experience different micro-environmental conditions and thus express a multitude of different physiologies and growth behaviors [42]. This growth pattern contributes sampling irregularities and certain variance in experimental measurements that prohibit absolute delineation of cellular responses to defined experimental conditions [43]; however, in this study we devised a number of measures into our experimental design for the consistent collection of reliable data. First, the synchronization of experimental cultures by age and growth stage was systematically confirmed by protein quantification and fluorescence microscopy. Repeated examination of *K. radiotolerans* cultures has proven protein quantification to be a reliable and reproducible method for inferring growth stage [10]. Furthermore, *K. radiotolerans* displays distinct growth stage-specific morphological transformations that provide independent validation of the protein-based assay (Figure 1). Second, the physiological and proteomic analyses performed in this study provide a collective measurement of enzyme activity, metabolite or peptide abundance at specific growth stages among replicate cultures (n = 3); thus, yielding a biochemical consensus of *K. radiotolerans* cells in response to an imposed environmental condition (i.e., Cu^2+^ concentration). Heterogeneity is unavoidable with a clumped culture, though the bio-molecular responses shown to have consistent and significant trends in activity or abundance should signify physiologically important functions promoting copper homeostasis in *K. radiotolerans.*

### Growth response to copper concentration

**Figure 2:**
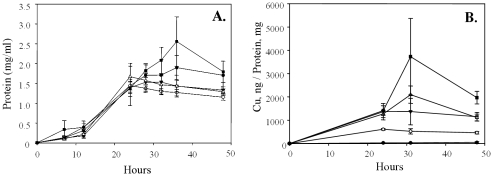
AB. Physiological response of *K. radiotolerans* to copper. Panel A: Growth response to increasing copper concentration. Panel B: Cytoplasmic accumulation of copper during growth. The experimental treatments are as follows; • TGY (No metal control), ○ 0.1 mM Cu(II), ▾ 0.75 mM Cu(II), Δ 1.0 mM Cu(II), ▪ 1.5 mM Cu(II).

**Figure 3:**
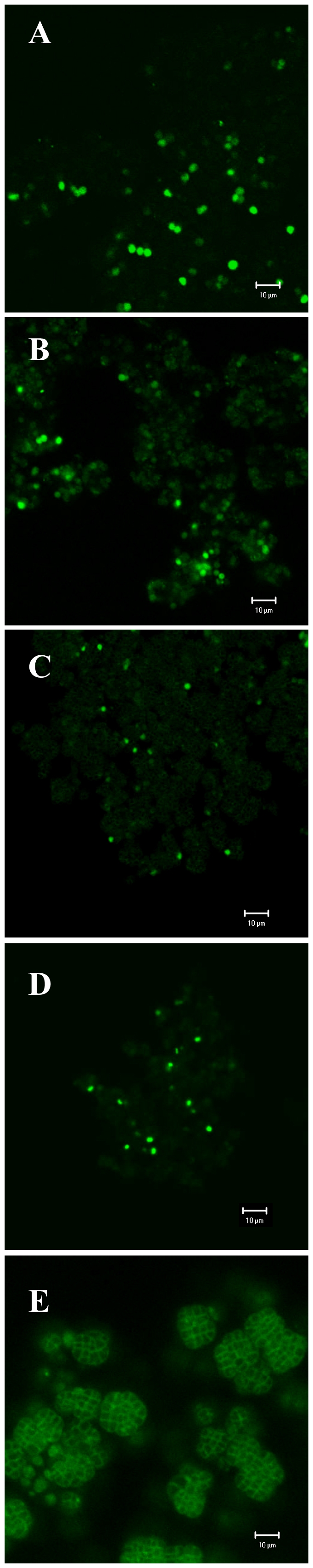
Fluorescent micrographs of reactive oxygen species (ROS, in green) in *K. radiotolerans* cultures (mid-exponential phase) as a function of copper concentration in the growth medium. A 10 µm scale bar is shown for each micrograph and lettered panel designations correspond to the following treatments: A.) No copper control, B.) 0.1 mM Cu(II) C.) 0.75 mM Cu(II), D.) 1.0 mM Cu(II), and E.) 1.5 mM Cu(II).

Triplicate cultures of *Kineococcus radiotolerans* were grown in TGY over a range of copper concentrations (0–1.5 mM) and cellular protein production was assayed at distinct growth phases. Overall, copper had no statistically supported affects on growth dynamics relative to the no-copper controls (Figure 2A). From these experiments, growth phases were operationally defined as follows; onset of exponential growth at 16 hr, mid-exponential phase at 22 hr, transition from exponential – stationary phase at 26 hr, and stationary phase at 32 hr. While not clearly evident from these results, cultures grown at the highest copper concentration (1.5 mM) displayed a marked increase in the extent of cell clumping (possibly flocculation; Figure 3) during active growth, which we presume could signify metal toxicity. Higher concentrations of copper (≥2 mM) were inhibitory to growth (data not shown).

### Intracellular copper accumulation

*K. radiotolerans* was previously shown to specifically and actively accumulate copper to high levels in the cytoplasm relative to other divalent cations, though only a single concentration was examined [10]. Here, intracellular contents were quantified over a range of copper concentrations at different growth phases (Figure 2B). There was a general trend towards increasing intracellular copper with increasing copper concentration in the growth medium (R^2^ = 0.7). Intracellular copper accumulation coincided with exponential growth and levels stabilized as cultures entered into stationary phase. Interestingly, the 1.0 and 1.5 mM Cu(II) grown cultures (2 out of 3 biological replicates) displayed an abrupt spike in intracellular Cu(II) levels during transition from late exponential growth into stationary phase (∼31 hr). While this result was statistically insignificant, a similar late-exponential phase increase in copper uptake has been demonstrated for *Streptomyces* spp. isolated from drainage channel sediments receiving copper contaminated discharge waters [30].

*K. radiotolerans* does not appear to excrete metal chelating siderophores in response to iron or copper as indicated by the lack of color reaction on modified chrome azurol agar plates (data not shown). This result is consistent with the apparent absence of siderophore biosynthesis genes from the genome, though putative siderophore interacting proteins and ABC-type transport components (Krad1239-41,-2670,-3704,-4105) have been annotated. Proteomics analysis, did in fact, reveal increased abundance of the ABC-type transporter encoded by Krad4105 from the 0.75 and 1.5 mM Cu(II) grown cultures at 16 hr and 32 hr (Table S1). Additionally, peptides for a putative isochorismatase hydrolase (Krad0796) were nearly 2-fold more abundant in the 1.5 mM Cu(II) grown cultures at 16 hr relative to the no copper controls. This protein can participate in siderophore biosynthesis, among other metabolic processes including hydrolysis of streptothricin antibiotics. The specific function of this or other putative siderophore interacting proteins was not empirically determined in this study.

Over the full range of copper concentrations, cultures were fluorescently stained at mid-exponential growth (22 hr) with a reactive oxygen species (ROS) sensitive dye and optically thin sectioned by confocal scanning laser microscopy (Figure 3). The reactivity of the assay was validated by exposing no copper, oxygen restricted control cultures to ROS inducer compounds; cell specific fluorescence increased demonstrably compared to the uninduced control cultures (data not shown). No obvious treatment effects were noted among the copper grown cultures ranging in concentration from 0–1.0 mM, and fluoresce was clearly cell specific. The 1.5 mM copper grown culture, however, displayed a strong uniform signal throughout entire cell aggregations; though fluorescence was predominantly associated with cell surfaces and the extracellular milieu and less from the intracellular environment.

Since cell growth (i.e., protein production) was similar up to 1.5 mM copper and the growth dependent copper accumulation profiles for the 0.75 and 1.0 mM treatments were nearly identical, subsequent experiments aimed to explore the biochemical responses invoked during intracellular copper accumulation only included copper at 0, 0.1, 0.75, and 1.5 mM (final concentration). The 1.0 mM Cu(II) treatment was omitted from further study.

### Copper-responsive proteome

Using the accurate mass and time (AMT) tag method of label-free quantitative proteome analysis a total of 1851 proteins were detected out of 4707 protein coding ORFs predicted from the *K. radiotolerans* genome from all time points (16, 22 and 32 hr) and growth conditions (0, 0.1, 0.75, or 1.5 mM Cu^2+^). Silva et al. [44] verified a relationship between MS signal response and protein concentration and that the average MS signal response for the three most intense tryptic peptides is constant within a coefficient of variation of +/−10%. Given this, we used spiked-in internal standards (both pre-digest and post-digest) to normalize all data runs and used the average of the top 3 most abundant peptides in all runs to calculate the average peptide response (relative abundance) for each observed protein in each LC-MS analysis. For this proteome discovery paper we included single peptide hits and proteins having only 2 peptide identifications for consideration in downstream biological interpretation with the caveat that protein abundance comparisons should be weighted less than the proteins identified with at least 3 peptides [45]–[46]. Of the 1851 proteins identified here, 345 were identified from 1 peptide and 203 were identified from 2 or more peptides. In all 1303 proteins were identified as having 3 or more unique peptides.

In our proteome analysis we calculated statistical significance of protein abundance changes separately for each copper concentration (0.1, 0.75, 1.5 mM) at each time point (16, 22, 32 hr) relative to the corresponding no copper control, thus yielding growth stage specific proteome responses to increasing concentrations of Cu(II) in the growth medium. ANOVA (p-value cutoff 0.01) was used to test for statistical significance which was operationally defined as a ±2 fold change in peptide abundance for at least one growth condition. In total, 1229 proteins met these criteria for a significant change in expression response to the imposed copper treatments.

**Figure 4:**
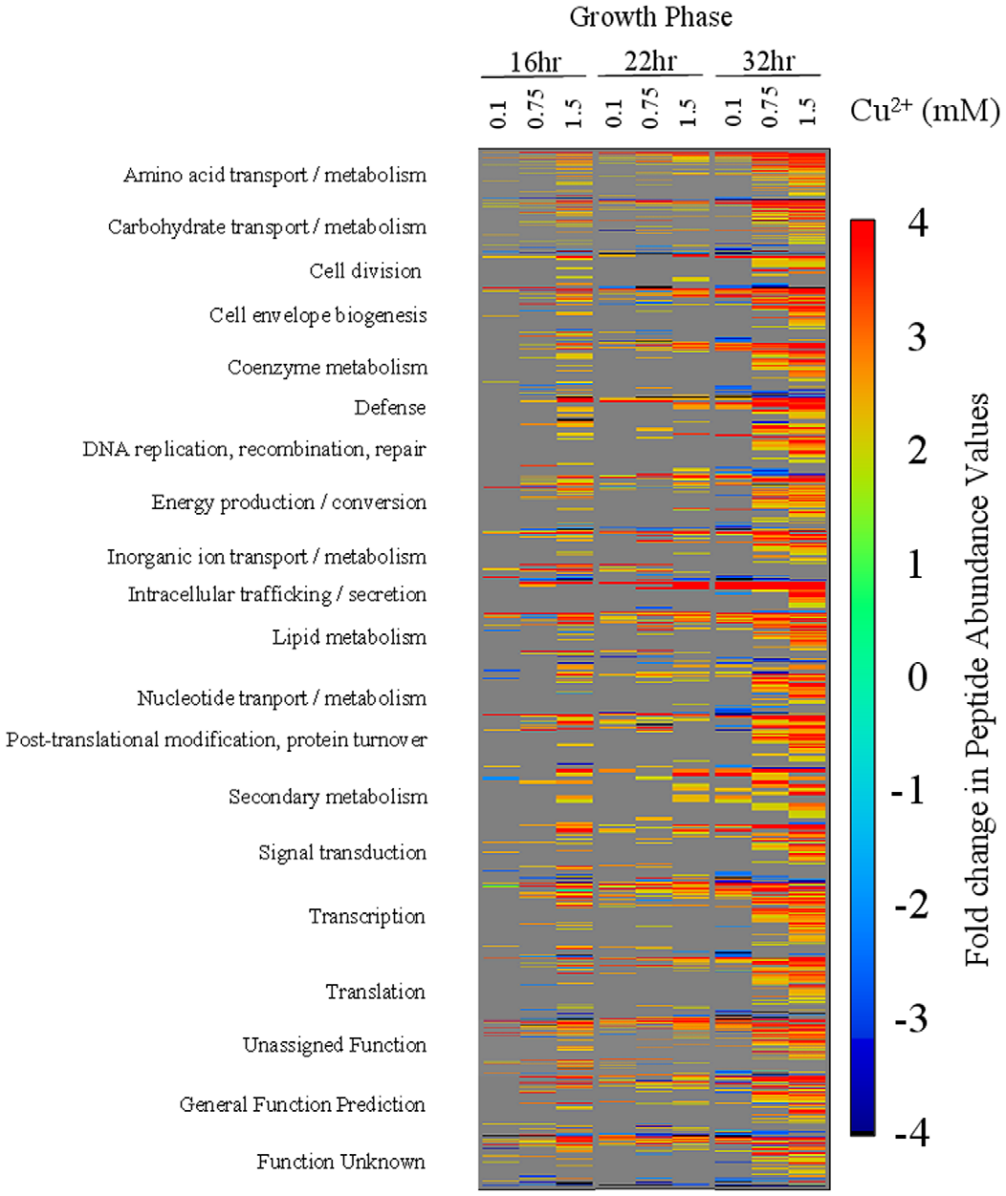
Composite heat map showing the proteomic response categories of *K. radiotolerans* to copper. Relative change in peptide abundance is shown for each of the copper growth conditions (0.1, 0.75, 1.5 mM Cu(II); the median response was calculated for biological triplicates) at a distinct growth stage (16 hr onset of exponential, 22 hr mid-exponential, 32 hr stationary phase) relative to the no copper controls.

A composite heat map of the copper responsive proteome of *K. radiotolerans* is shown in Figure 4. Overall, the majority of differentially expressed peptides displayed an increase in expression that generally corresponded with growth phase and copper concentration; most notably the 0.75 and 1.5 mM Cu(II) grown cultures at stationary phase (32 hr). Exceptions include inorganic ion transport and metabolism and unassigned COG categories. Expression patterns for these functional categories showed more differences in response proteins between treatments and revealed proteins whose expression was more sensitive to low-moderate copper concentrations. Whole-cell proteomic responses of key functional categories are provided in detail in the supplementary section (Tables S1, S2, S3, S4, S5, S6 and S7).

### Central metabolism and energy production

Numerous proteins participating in glycolysis and gluconeogenesis, TCA cycle, pentose phosphate and interconversion pathways were identified, as well as glycogen debranching enzymes. Increased expression of proteins involved in biosynthesis of all essential amino acids (including pyruvate metabolism and NAD^+^ cofactor synthesis), conversion of nonessential amino acids, and ammonia scavenging via the urea cycle were measured. Lastly, all necessary components for replication (DNA Pol I and III), transcription, aerobic electron transfer and ATP production were identified [47]. Protein abundance increased for Krad1604, a putative polysulfide reductase (NrfD), at 22 hr for the 0.75 (2.4 fold change) and 1.5 mM (2.5 fold change) Cu(II) grown cultures and for all copper treatments by 32 hr (2.4, 9.5, 9.3 fold change for the 0.1, 0.75, and 1.5 mM treatments, respectively). Enzymes belonging to this pathway fulfill versatile roles in cellular physiology, principally energy conversion, but participation in the maintenance of cellular redox balance [48] and pH homeostasis [49] has also been demonstrated. Concomitant expression of putative formate dehydrogenases (Krad1602, 3339: 3.0 fold change at 32 hr for 0.75 and 1.5 mM treatments), which couple formate oxidation to the reduction of NAD^+^ or cytochromes, lends additional weight to a stress response interpretation; formate has been shown to extend the viability of *K. radiotolerans* during prolonged starvation [47]. A suite of transcriptional regulators were also detected (differential expression was not significant); TetR family (Krad2707,-1300, -4318, -0267, -2116), MarR (Krad2043, -3930, -2941, -2894, -4251), DeoR family (Krad0304, -1535), GntR family (Krad4211, -1921, -2681). Overall, surprisingly few proteins were less abundant in the experimental cultures relative to the no copper controls (Krad2981, 2945, 3852, 3159, 1254), and no clear response or functional pattern could be deduced from these proteins collectively.

### DNA repair and chromosomal maintenance

We presume that the copper treatments affected the incidence of DNA damage during growth of *K. radiotolerans* as evident by the abundance of peptides participating in damage specific repair pathways, as well as members of the SOS regulon (Table S2).

Several proteins detected were annotated for nucleotide excision repair (NER), including; a putative DNA-dependent ATPases (Krad1067) for transcription-coupled DNA repair [50], an exinuclease ABC, B subunit (Krad2942), a transcription repair coupling factor helicase (Krad0167), and an UvrD/REP type DNA helicase potentially involved in DNA stabilization and repair (Krad1179). Krad3140, the only MutT homolog detected out of the 5 annotated alleles present on the genome, encodes for 8-oxo-GTPase (prevents misincorporation of oxidized dGTP), whose peptides were numerically abundant at the highest copper concentrations during stationary phase.

Base excision repair was also expressed; proteomics analysis detected uracil-DNA glycosylase (Krad3639), peptide abundance increased more than 2-fold in the 0.1 and 1.5 mM Cu(II) growth conditions. Also detected from the 1.5 mM Cu(II) grown culture (32 hr) was an exodeoxyribonuclease III Xth (Krad3979), an endonuclease of apurinic/apyrimidinic (AP) sites, an ATP-dependent DNA ligase (Krad0653) and DNA polymerase I (PolA; Krad2951).

Induction of the SOS response was evident by whole cell proteomics analysis. Peptides detected include RecA (Krad1492) for recombinational DNA repair, along with participant proteins RecF (Krad0004), RecG (Krad1368- at the highest copper concentration at all times and 32 hr for all copper treatments), RecN (Krad3147), RecQ (Krad4305; ATP-dependent DNA helicase), and RecR (Krad0465). Concomitant expression of DNA-directed RNA polymerase [α (Krad0715), β (Krad0680), Ω subunits (Krad2991)] was also measured from the highest copper treatments (0.75 and 1.5 mM). Krad0757 (HelD) helicase IV, which participates in the RecF pathway of recombination, was abundant at 22 hr for the 1.5 mM Cu(II) treatment and for all other copper treatments by 32 hr. The RuvB helicase subunit of the RuvABC resolvasome (Krad3828) was also detected; this enzyme is involved in the latter stages of homologous recombination. LexA (Krad1506) peptides were detected (differential expression was not significant) but only at 32 hr from the 0.75 and 1.5 mM Cu(II) grown cultures.

Finally, Krad1121, an exodeoxyribonuclease VII involved in the degradation of single-stranded DNA or perhaps damaged nucleic acids, along with a helicase (Krad3612), were detected at 32 hr in the 0.75 mM and 1.5 mM Cu(II) grown cultures. We also detected peptides for Krad0603, a putative RadA. Interestingly, RadA is essential for radiation resistance in *Escherichia coli* and *Deinococcus radiodurans* R1 but unnecessary for peroxide resistance [51], [52]. Krad4333, a replicative DNA helicase, was detected for each of the copper treatments but inconsistently among the biological replicates. Enzymes involved in the biosynthesis of carotenoids (phytoene synthase, Krad3229, and phytoene desaturase, Krad3225, 3228) were detected only at 16 hr of growth from the 1.5 mM Cu(II) grown cultures. Numerous DNA binding proteins were detected from each of the Cu(II) grown cultures; including DNA binding ferritin Dps family proteins (Krad1159,4280) which possess a ferroxidase center for metal sequestration and has been shown to protect DNA against oxidative damage [53]. Several additional nucleic acid binding proteins were detected at various sampling points for the highest copper treatment (1.5 mM); putative nucleic acid binding proteins (Krad1551, 4509, 4338), DNA topoisomerase I (Krad0487, TopA) a DnaK chaperone (Krad4233, at 32 hr), HupB/IHF DNA binding proteins (Krad1360, -2805, -2005) were abundant for the 0.75 and 1.5 mM Cu(II) grown cultures, and lastly, histone-like proteins (Krad1306,3371). Xanthosine triphosphate pyrophosphatase HAM1/YggV (Krad3762) was abundant at all sampling points for the 1.5 mM Cu(II) grown cultures and was strongly up-regulated by 32 hr for all other copper treatments.

### Stress Proteins

A variety of stress proteins were differentially expressed in response to the copper supplemented growth conditions; heavy metal transporters or efflux proteins (Krad0783,1116, 1868, 3308, 3705), drug resistance exporters (Krad1480,3923, 3287,0860,1284,0951), general stress proteins (Krad0404,0263,3309,3308), heat and cold shock proteins (Krad3580,0218,0353,3637), and a heat inducible transcriptional repressor HrcA (Kard3406). Heat shock proteins are responsive to treatments that result in protein level damage, facilitating protein refolding or degradation of irreversibly damaged proteins [54]–[57]. Interestingly, the abundance of peptides for a putative copper resistance protein (Krad4275) did not correlate with copper concentration; In fact, peptides were significantly less abundant in the 0.75 mM Cu(II) grown culture by 32 hr (−5.6 fold change) than in the no copper controls.

### Oxidative stress defenses

Copper enhanced production of reactive oxygen species (ROS) was evident from the 1.5 mM Cu(II) grown cultures (Figure 3), though it remained unclear whether there was a significant oxidative response at lower concentrations of copper. Laboratory assays were used to carefully measure the activity of specific oxidative defense enzymes expected to have an important role in the cellular response to copper. Whole-cell proteomics provided an unbiased description of additional participatory proteins that may help defend the cell against copper's reactivity. Combined, these approaches should provide a reliable indication of how copper supplementation, and thus cytoplasmic accumulation, affects oxidative stress levels in *K. radiotolerans*.

**Figure 5:**
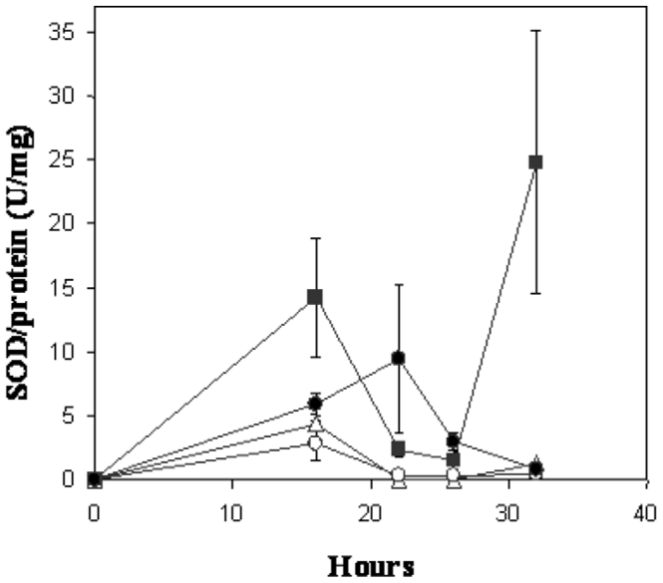
Superoxide dismutase activity profiles during the growth of *K. radiotolerans* in copper supplemented medium. Cultures (n = 3) were grown in TGY medium amended with CuSO_4_ and incubated at 28°C and 150 rpm. Growth phases were operationally defined as follows; onset of exponential growth at 16 hr, mid-exponential phase at 22 hr, transition from exponential – stationary phase at 26 hr, and stationary phase at 32 hr. The experimental treatments are as follows; • TGY (No metal control), Δ 0.1 mM Cu(II), ○ 0.75 mM Cu(II), ▪ 1.5 mM Cu(II).

Superoxide dismutase (SOD; EC 1.15.1.1) catalyzes the dismutation of superoxide anions to hydrogen peroxide and molecular oxygen, and the *K. radiotolerans* genome encodes a single putative Fe/Mn-containing SOD (Krad3578). Superoxide anion poses a relatively minor threat for causing direct damage to DNA [55]. Its toxicity centers on its reactivity with redox active metals (i.e., copper) resulting in the production of peroxides and hydroxyl radicals via Fenton chemistry [58]. In the no copper control cultures, SOD activity corresponded with bacterial growth; increasing consistently to around 22 hr of exponential growth followed by a sharp decline as the culture entered stationary phase (Figure 5). Proteomics analysis did not reveal a significant treatment response in the abundance of peptides for SOD, though enzyme assays suggest that activity responded to copper supplementation in two different ways. At 0.1 and 0.75 mM Cu(II), SOD activity was incrementally lessened relative to the no copper controls. Enzyme activity increased, though only slightly, up to 16 hr of cell growth (onset of exponential phase) and then declined to non-detectable levels through 26 hr; beyond 32 hr, SOD exhibited a low level of activity. Conversely, at 1.5 mM Cu(II), SOD activity increased immediately and rapidly through early exponential phase (16 hr) followed by a sharp decline to a low level of activity until 28 hr; whereupon the transition into stationary phase signified another sharp spike in SOD activity.

A series of experiments were performed to further evaluate the apparent copper responsive down-regulation (mid-exponential) and rebound (stationary phase) of SOD activity in *K. radiotolerans*. First, we determined whether SOD activity could be induced in copper grown cultures by intentional exposure to superoxide anion. Consistent with previous results, SOD activity was not detected during mid-exponential growth from 0.75 mM Cu(II) grown cultures (data not shown). SOD activity remained below detection following incubation with paraquot, a known catalyst for superoxide anion [59] used alone and in combination with excess copper. Paraquot treatments generated a significant increase in SOD activity (50 U/mg) from the no copper control cultures. In parallel, Cu(II) grown cultures (0.75 mM) were pre-treated with chloramphenicol (a protein translation inhibitor); we not only reproduced the rebound in SOD activity during stationary phase, but activity was responsive to combined treatments of paraquot and copper (i.e., activity nearly doubled over controls).

These results are not conclusive, but may imply that copper (0.1–0.75 mM) influences the post-translational repression of SOD during exponential growth of *K. radiotolerans*. Numerous studies have documented the coordinated expression of SOD isozymes in response to heavy metal toxicity [60]–[61] and reactive oxygen species [59], [62]. Schmidt et al. [35] demonstrated heavy metal induced transcriptional repression of the FeSOD for a metal resistant streptomyces isolate with concurrent expression of the nickel containing isozyme NiSOD (*sodN*). The *K. radiotolerans* genome has been annotated for only a single isozyme (Fe/Mn) and putative NiSOD homolog queries have returned no hits. Repression of SOD activity in response to copper was unexpected considering that *sod* knockouts in *S. cerevisiae* markedly increases sensitivity to copper and oxygen [60]. At low to moderate copper concentrations, strict regulation of SOD could mitigate production of stronger oxidants within the cell [63]. SOD did, however, display a considerable response in cultures grown at 1.5 mM copper, suggesting an important role against copper toxicity at sub-lethal concentrations.

Catalase (EC 1.11.1.6) catalyzes the decomposition of hydrogen peroxide to water and oxygen without the production of free radicals. The *K. radiotolerans* genome encodes a putative Mn-containing catalase (Krad0815; COG3546) and a KatE-like catalase (Krad0865; COG0753); as well as several presumptive peroxidases (Krad1247,-3350) and redoxin-type antioxidants (Krad0803,-0848,-1178,-1282,-2298) whose activities could potentially react with the assay used in these experiments. Catalase activity was consistently detected for all treatments though averaged activity was generally low (2 U/mg) and variability was too high to reveal any trends (data not shown). Direct comparison of our data with the literature is problematic on account of differences in methodology, though these values are likely well below previous measurements for wild-type *E. coli* [64], and more closely approximate that of catalase-impaired mutants [65]. Proteomics analysis only detected 1 allele (Krad0815) at 32 hr, but these peptides were significantly less abundant for the 0.1 (−6.3 fold change) and 0.75 mM (−3.7 fold change) Cu(II) grown cultures than the no copper controls. Catalase is largely regarded as a less informative diagnostic enzyme than inducible peroxidases which can be more finely attuned with environmental conditions [63], [66], [67]. Indeed, peptides for putative thioredoxins (Krad0848, 4504) and a thioredoxin reductase (Krad4503) were significantly up-regulated for the 0.75 and 1.5 mM Cu(II) cultures at 16 hr and 32 hr. This antioxidant facilitates protein reduction through cysteine thiol-disulfide exchange reactions, and can serve as an electron donor for peroxidases. Additional copper responsive oxidative defense enzymes detected by whole cell proteomics analysis includes a plasmid encoded DSBA oxidoreductase (Krad4572), a member of the thioredoxin family that catalyzes disulfide bonding and proper folding of secreted proteins. This periplasmic protein is involved in the pathogenesis of *Haemophilus influenzae* [68], toxin production in *Vibrio cholerae* [69], and intestinal colonization of adherent-invasive *E. coli* (AIEC) in Crohn's disease [70]. Its potential role in this Gram positive actinobacterium is unknown but most likely involved in oxidative stress response. Also detected was an FAD-dependent pyridine nucleotide-disulfide oxidoreductase (Krad3918) which has been linked to the reduction of epoxides in *Xanthobacter* [71]. Other notable enzymes detected include a chloride peroxidase (Krad0128), catalyzes the decomposition of hydrogen peroxide, and methionine sulfoxide reductase (Krad 1091, MsrA), functions in the repair of oxidized proteins; peptides for each were detected from the 0.75 and 1.5 mM Cu(II) grown cultures at 22 hr and 32 hr (Table S3). OsmC family proteins (Krad0349,-0859, and -3648) are regarded as stress-induced, detoxification proteins in *E. coli*; peptides for these proteins were abundant (2–3 fold change) by 32 hr for cultures grown in 0.75 and 1.5 mM Cu(II) supplemented growth medium. Peptides for an organic hydroperoxidase (Krad0813) were only detected at 22 hr for all of the copper treatments, as well as a Dyp-type peroxidase (Krad3350) and an alkyl hydroperoxide reductase (AhpC; Krad3757) which directly reduces organic hydroperoxides; though differential expression levels were not statistically significant.

### Antioxidants and protective metabolites

**Figure 6:**
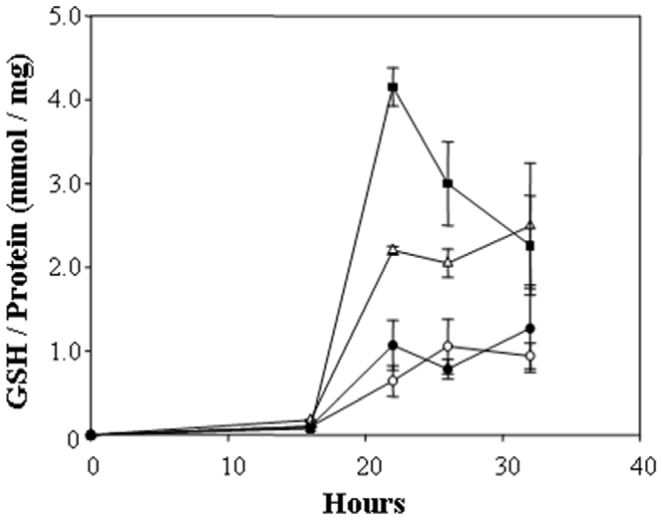
Glutathione levels during the growth of *K. radiotolerans* in copper supplemented medium. Cultures (n = 3) were grown in TGY medium amended with CuSO_4_ and incubated at 28°C and 150 rpm. Growth phases were operationally defined as follows; onset of exponential growth at 16 hr, mid-exponential phase at 22 hr, transition from exponential – stationary phase at 26 hr, and stationary phase at 32 hr. The experimental treatments are as follows; • TGY (No metal control), Δ 0.1 mM Cu(II), ○ 0.75 mM Cu(II), ▪ 1.5 mM Cu(II).

The tripeptide glutathione can potentially fulfill two important cellular functions; metal sequestration and oxidative detoxification [54]. Analysis of the *K. radiotolerans* genome revealed a putative glutathione peroxidase (Krad1247; EC 1.11.1.9), a glutathione S-transferase (Krad0366), and a glutathionylspermidine synthase (Krad2757; EC 6.3.1.8); however, homologs for GSH synthesis (glutamylcysteine synthetase, GSH synthetase) and a glutathione reductase could not be identified. Proteomics analysis did not detect peptides for any of the proteins involved in glutathione metabolism. Total glutathione (GSH + GSSH, GS-Cu) was monitored from triplicate cultures at distinct growth phases in response to increasing copper concentration (Figure 6). Glutathione production corresponded with active cell growth for all treatments; levels peaked at 24 hr of growth and stabilized, except at the highest copper concentration (1.5 mM) where levels decreased significantly. No copper controls and cultures grown at the moderate copper dose (0.75 mM) maintained nearly identical GSH levels over the course of the experiment, while *K. radiotolerans* produced significantly more GSH in growth medium containing 0.1 mM Cu(II). The highest levels of GSH were produced and sustained at the 1.5 mM Cu(II) growth condition. GSH production was also responsive to environmental conditions intended to produce oxidative radicals; levels doubled upon treatment of 0.75 mM Cu(II) grown cultures with paraquot (data not shown).

While we did not specifically measure antioxidative cellular capacity or the accumulation of other stress induced compatible solutes, whole-cell proteomics analysis detected peptides for the biosynthesis of glutamine, glutamate, non-reducing saccharides (trehalose; Krad0920, -3747 and maltooligosyl trehalose, Krad3075) and the conversion of glycogen to trehalose (Krad3074). Enzymes involved in the biosynthesis of trehalose were detected in *K. radiotolerans* cultures in response to copper; however expression levels failed to meet our ±2 fold cutoff for statistical significance. Trehalose accumulation in response to multiple stresses, including copper sulfate [72], has been demonstrated in many bacterial systems and this proteomic snapshot suggests the co-expression of two distinct trehalose biosynthesis pathways in *K. radiotolerans* [73]. Peptides for Krad2708 and -4349 were significantly more abundant at the highest copper concentrations (0.75 and 1.5 mM Cu^2+^) relative to the controls. Both ORFs encode for a putative 2,5-didehydrogluconate reductase which participates in the production of ascorbate and thus may regulate intracellular redox conditions. The substrate binding region of an ABC-type glycine betaine transport system (Krad1293) was strongly up-regulated at mid-exponential growth and continued into stationary phase in the copper grown cultures. If accumulated, glycine betaine could also help boost antioxidative defenses [74]. Numerous ORFs catalyzing the production of terpenes and terpenoids were also detected (though differential expression was not significant); these compounds have been implicated for having anti-oxidative defense properties as well [75].

### Post-translational modification and protein turnover

Copper enhanced production of ROS could directly produce protein damage. GrpE (Krad4232) and DnaJ (Krad4231) were significantly up-regulated for the 0.75 and 1.5 mM Cu(II) grown cultures and at all copper concentrations at 32 hr. These gene products are co-chaperones for DnaK which is involved in intracellular housekeeping and stress functions. In *E. coli,* GrpE participates in osmotic and heat shock by preventing aggregation of stress-denatured proteins, as well as the degradation of unfolded proteins. Other copper responsive proteins detected include Krad2917, encodes for SufB, which is part of the SufBCD complex that contributes to the assembly or repair of oxygen-labile FeS clusters following oxidative stress. Krad1873 and -1874 encode for 20S proteasome A and B subunits, respectively, and are involved in the degradation of damaged proteins by compartmentalized proteolysis [76].

### Sporulation

*K. radiotolerans* is not known to produce spores, and closely related species are non-spore forming [12], [77], though complex multi-structural formations are typical for actinomycetes during distinct life-cycle or developmental stages. The 0.75 mM Cu(II) grown cultures displayed strong expression of a putative spoOM protein (Krad0063) at 32 hr, which has been shown to exert certain control over sporulation in *Bacillus subtilis* [78]. In addition, peptides for Krad0122 and -3698, annotated as stage II sporulation E family proteins, were both consistently abundant in cultures grown at 0.75 and 1.5 mM Cu(II). Krad2323 and -3437 peptides were also detected; both loci are annotated as LuxR homologs. LuxR has been extensively characterized as the transcriptional activator for a multitude of cooperative behaviors in bacteria, the best example being bioluminescence in *Vibrio fischeri* [79]. Perhaps related, the expression of various luciferase family proteins (Krad3320, -3294, -3725, -3286, -0961) was also detected among the Cu(II) grown cultures, however the function of these gene products is unknown. Lastly, peptides for Krad1390 increased at 32 hr in the 0.75 and 1.5 mM copper treatments; this ORF encodes a putative SMC chromosome structural protein that may be involved in chromosomal condensation or possibly life-cycle responsive organization.

In *K. radiotolerans,* intracellular copper accumulation increased with concentration without measurable negative affects on cell growth, biomass production, or detectable levels of intracellular oxidant. Copper must be actively and efficiently sequestered once it enters the cell and while this study revealed few clues about the intracellular fate and trafficking of copper (several metal binding proteins were highlighted as promising targets for future research), capacity and maintenance of a reduced intracellular environment would favor metal sequestration [57], [80] as well as preserve critical cell functions. The importance of a strictly maintained intracellular environment, particularly at higher concentrations of copper, is underscored by the strong up-regulation and abundance of proteins having annotated functions for the binding and stabilization of nucleic acids and proteins and presumptive metabolic flux towards production of reductants, potent antioxidants, and protectant metabolites. As expected, we observed direct up-regulation of various enzymatic and nonenzymatic defenses at the physiological limits for copper clearly suggestive of a predominant metal stress or toxicity response; however cell staining for reactive oxygen species showed no cell specific fluorescence as was observed at lower copper concentrations (0–1.0 mM Cu^2+^) but rather only uniform fluorescence of the thick extracellular matrix. This result could imply the extracellular activity of antioxidant enzymes, enhanced scavenging of reactive oxygen species by the EPS [81], or perhaps chain reactions propagated by reactive moieties (e.g., adsorbed copper, lipid peroxidation). Conversely, low to moderate copper concentrations appear to induce condition-specific response pathways that are not yet fully understood because of the strong response of weakly annotated COG categories (accounting for nearly 25% of all differentially expressed proteins). Development of a reliable genetic system is the next step in properly evaluating the functionality of these proteins and their role in promoting stress resistance and copper homeostasis.

## Materials and Methods

### Culture conditions and chemicals

*Kineococcus radiotolerans* (BAA-149) was obtained from the American Type Culture Collection (ATCC; Manassas, VA, USA). Cultures were grown on TGY medium (1.0% tryptone, 0.5% yeast extract, 0.1% glucose) at 28°C and shaken at 150 rpm for liquid cultures unless otherwise stated. Frozen cultures were prepared using the Microbank™ Bacterial Preservation System (Pro-Lab Diagnostics, ON, Canada) and were stored at −80°C. Stock solutions of copper sulfate, paraquot, and chloramphenicol were prepared fresh in deionized water, filter sterilized and stored at 4°C.

### Growth response to copper

*K. radiotolerans* was inoculated (2.0% v/v) in triplicate into TGY amended with 0.0 (control), 0.1, 0.75, 1.0 and 1.5 mM cupric sulfate. Cellular growth was evaluated by protein quantification using the *DC* Protein Assay Kit (Bio-Rad, Hercules, Calif.). Total intracellular metal contents were quantified by inductively coupled plasma-mass spectroscopy (ICP-MS) at distinct phases of growth. Briefly, cells were harvested by centrifugation (10,000× g, 5 min, 15°C) and sequentially washed 3x each in 50 mM EDTA/1x PBS (pH 7.5; 1xPBS = 137 mM NaCl, 2.7 mM KCl, 10 mM Na_2_HPO_4_, 2 mM KH_2_PO_4_) and 25 mM EDTA/1x PBS in order to remove weakly cell surface adsorbed metal. Cells were then washed 3x in PBS and immediately frozen in liquid nitrogen and stored at −80°C. The effectiveness of these metal chelation wash steps has been confirmed [10]. For intracellular metal analysis, cell pellets were digested at room temperature in 0.1 mL ACS grade concentrated H_2_O_2_ and 0.2 mL concentrated optima grade HNO_3_. The samples were then diluted with 18.2 MOhm*cm DI water and analyzed in standard mode on a Perkin Elmer-Sciex Elan DRC Plus ICP-MS according to EPA method 6020a. External calibration was performed using NIST traceable standards diluted in the same matrix as the samples and the calibration was verified against a standard with a different lot number.

### Oxidative Stress and Confocal Laser Scanning Microscopy

In conjunction with the growth experiments described above, copper grown *K. radiotolerans* cultures were examined for relative levels of oxidative stress using the Image-iT™ Live Green Reactive Oxygen Species Detection Kit (Molecular Probes, Eugene OR USA) by confocal laser scanning microscopy (LSM). The Image-iT™ Live Kit was used per the manufacturer's instructions, though optimum cell staining was achieved by reducing the incubation to 5 min. Cell preparations were viewed with SlowFade™ (Molecular Probes) and x-,y-,z- scans were conducted at 0.7 µm increments using a 510 LSM (Carl Zeiss Inc., Thronwood, N.Y.). Control cultures were grown in capped serum bottles with the headspace flushed with nitrogen gas to reduce oxygen exposure (negative control) or were exposed to reactive oxygen species (ROS) inducer compounds *tert*-butyl hydroperoxide (TBHP) and paraquot (positive controls).

### Copper transport

A modified chrome azurol S (CAS) agar plate assay was conducted for siderophore production as described in Milagres et al. [82] using either 1.0 mM (final concentration) Cu(II) (as CuSO_4_) or Fe(II) (as FeCl_3_·6H_2_O). *Shewanella oneidensis* MR-1 (BAA-1096) was used as a positive control strain for siderophore production, and *D. radiodurans* (BAA-816) was selected as a negative control strain [83]. Concentrated hydrochloric acid was used as an abiotic control to ensure reactivity of the CAS agar plates.

### Biochemical assays

Cultures were sampled (1 mL) at specific times and growth phases using large bore tips. Briefly, cells were harvested by centrifugation (10,000× g, 3 min, 4°C) and washed subsequently with ice cold metal chelation buffer as described above to remove extracellularly bound copper. Cell lysis was accomplished by horn sonication (maximum power setting) in an ice bath for 2 minutes followed by bead beating with 2 µm glass beads for 2 cycles at one minute each. Cell lysates were used for the following biochemical assays per the manufacturer's instructions: superoxide dismutase (SOD) (Trevigen, Gaithursburg, MD), catalase (Biomedical Research Service Center, NY), and glutathione (GSH) (Norwthwest Life Science Specialities, LLC, WA). All assays were quantified by spectrophotometry and experimental values determined by linear regression of known standards provided with the kits (SOD, GSH) or purchased separately (catalase; Fisher Scientific, NJ). Means and standard deviations were calculated from experimental replicates (n = 3); enzyme activities (U/mL) and GSH levels (mMoles/mL) were normalized by cellular protein (mg/mL).

### SOD regulation

Experiments were devised to delineate SOD activity patterns and the potential mode of copper-dependent regulation. Parallel *K. radiotolerans* cultures (2.0% v/v inoculum from an exponentially grown mother culture; n = 3) were grown to early exponential phase (22 hrs) in TGY with 0.75 mM copper or in TGY with no added copper. Cells were harvested by centrifugation, washed in 1x PBS (pH 7.0), and inoculated into fresh medium (n = 3) with and without paraquot (200 µM, final concentration), a known producer of superoxide anion, and additional copper (0.75 mM, final concentration). Cultures were incubated for 4 hrs at 28°C and 150 rpm. SOD activity was measured as described above. Secondly, *K. radiotolerans* cultures were grown to late exponential phase (26 hrs) in TGY with 0.75 mM copper. Cultures were spiked with treatment combinations of chloramphenicol (50 µg/mL) and additional copper (0.75 mM), incubated for 4 hrs at 28°C and 150 rpm, and SOD activity was measured.

### Proteome preparation

The following chemicals used, unless otherwise noted, were obtained from the Sigma-Aldrich Company (St. Louis, MO) and were of analytical grade. Flash frozen cell pellets were lysed in nanopure water using 0.1 mm silica beads and a mini-bead beater (Biospec, Bartlesville OK) for 90 s at 4500 rpm. Lysates were placed immediately on ice to inhibit proteolysis. The protein concentration of each whole cell lysate was measured using a Coomassie Plus protein assay (Pierce, Rockford, IL) using a bovine serum albumin standard. The internal protein standard consisted of 1 mg of apomyoglobin (equine), 0.363 mg of cytochrome c (bovine) and 0.210 mg of G-2-PHD (rabbit) in 1 mL (final volume) of nanopure water. To each whole protein sample, supernatant (soluble protein) fractions and pellet (membrane protein) fractions, 10 µL (15.73 µg) of the internal protein standard was added to every 984 µg of sample.

The whole cell lysates were trypsin digested and then desalted using Supelclean C-18 tubes (Supelco; St. Louis, MO) as described by Masselon et al. [84] with the exception that the peptides were alkylated in the presence of 20 mM iodoacetamide for 30 min at room temperature. Eluted peptides were concentrated via speedvac (ThermoSavant, San Jose CA) and peptide concentrations were determined by BCA assay (Pierce, Rockford IL) with a bovine serum albumin standard. Protein concentrations were standardized to 1.0 mg/mL. The yeast alcohol dehydrogenase pre-digested mass spectrometry standard (MassPREP ADH Digestion standard, Waters Corporation, Milford MA) was added to each sample (50 fmol/µg peptide).

### SCX Fractionation of Peptides for Potential Mass and Time (PMT) Tag Acquisition

300 µg sample pools with equal amounts of peptide mass were combined from all 16 hr, 22 hr and 32 hr samples. These pooled samples were separated with a strong cation exchange (SCX) fractionation as described elsewhere. [85].

### Capillary LC Separations

All peptide mixtures from the whole cell lysate SCX fractions were separated by an automated custom designed HPLC system [described in detail elsewhere; 84,86] and eluted directly into either an ion trap (MS/MS) or an Orbitrap MS.

### Peptide Mass and Time Tag (PMT) Acquisition

The eluate from the HPLC was directly electrosprayed into an ion trap MS (LCQ, ThermoFinnigan, San Jose, CA) using electrospray ionization (ESI). The mass spectrometer was operated in a data-dependent MS/MS mode and analyzed with one full *m*/*z* range (400–2000) each. The details for PMT generation are described elsewhere [87]. The MS/MS spectra were analyzed using the peptide identification software SEQUEST [88] in conjunction with the annotated protein translations from the genome sequence of *K. radiotolerans* SRS30216 (http://www.genome.jp/kegg/kegg2.html, NC_009664, NC_009806, and NC_009660) using a +/−2 Da tolerance for precursor ions and a +/−0.8 Da tolerance for MS/MS fragment ions. A dynamic modification search (i.e., the presence and absence of the modification was searched) for methionine oxidation and a static search (i.e., presence of the modification was searched only) for alkylation of the cysteines by iodoacetamide. Non-enzyme cleavage constraints were applied. Qualitative/cursory identifications in the putative mass and elution time (PMT tag) peptide library were based on a minimum cross correlation (Xcorr) score of 2 for all peptides identified at least twice in all MS/MS experiments. P-values and error rates associated to the peptide identifications were calculated as reported previously [89]. A p-value cutoff of 0.01 related to an estimated false discovery rate of 1% was applied to the MS/MS identifications.

### Accurate Mass and Time (AMT) Tag Identification and Alignment

The method used here for label-free quantitative proteomics is known as the AMT tag method [90]. Using 5 µg (0.5 µg/µL) of total peptide from each of the samples analyzed above by MS/MS, intact peptide mass data were obtained by HPLC equipped with a Thermo Finnigan Orbitrap mass spectrometer. The run order was randomized in a Latin Squares design. A mass calibration mixture was infused at the end of each analysis, and the masses of the compounds in the mixture were used to calibrate the spectra. Data was collected for 100 minutes, beginning 65 minutes after the sample was injected (15 minutes into gradient). Orbitrap spectra (AGC 1×10^6^) were collected using a full range scan of 400–2000 m/z at a resolution of 100k followed by the data dependent ion trap MS/MS spectra (AGC 1×10^4^) of the three most abundant ions using a collisional energy of 35%. Previously analyzed ions were excluded using a dynamic exclusion time of one minute.

The unique peptides (trypsin cleaved peptide sequence unique to one protein) detected by the LTQ ion trap mass spectrometer were matched with those identified as PMT tags. The data were subsequently processed using the PRISM Data Analysis System, a series of software tools developed in-house. The data were initially de-isotoped to give a monoisotopic mass, charge, and intensity of the major peaks in each spectrum. The data were then analyzed in a two-dimensional fashion to determine the groups of peaks that were observed in sequential spectra.

Each group, identified as a unique mass class (UMC), was characterized as having a distinctive median mass, central normalized elution time (NET), and abundance estimate, calculated as the sum of the intensities for the MS peaks of that UMC. Each UMC was determined by comparing the mass and NET to those in the PMT tag database that passed the p-value cutoff of 0.01 using the in-house developed software Multialign (http://omics.pnl.gov/software). Search tolerances were set to ±1.5 ppm for the mass and ±1.5% for the elution time. Those UMCs that most closely matched the PMT tags were validated as Accurate Mass and Time (AMT) tags; moreover, this technique provided a list of peptides observed and an abundance value for each. The abundance value was calculated as the peak height of the ion current chromatogram of the eluting peptide for the most abundant charge state.

### Normalization of Replicate Analyses

Identified and validated peptides were imported into an Excel file. For protein (rollup) identification in each LC-MS run the average of the top 3 most abundant and most present peptides (calculated by multiplying the number of runs the peptide was identified by the average value seen in all runs) was calculated to confer the protein abundance value [44]. Proteins with only 1 or 2 peptides identified were also included for consideration but the number of peptides identified for each protein is included in all supplementary data tables. Each LC-MS run was normalized against the average value of all pre-digestion internal standards averaged with the post-digestion standard value. These internal standard normalized protein values were then taken into the in-house developed software DAnTE (http://omics.pnl.gov/software/) [91] which was developed for downstream data transformation, normalization and visualization. In DAnTE, all data from each dataset were converted into a log base 2 value and each of the triplicate instrument replicates of the same sample were then linear regression normalized using a reference line composed of values obtained from the median value obtained from each of the three replicates. These abundance values were made into a text file and imported into the text file and imported into MeV (http://www.tm4.org). Here a one way ANOVA statistical test was performed on each dataset for each time point (16, 22, or 32 hr). In all 1367 *K. radiotolerans* proteins passed the p-value cutoff filter of 0.01 in at least one of the ANOVA tests. Fold changes relative to the median abundance value of the 0 µM Cu(II) growth condition for each time point (i.e., 16 hr data was only compared to itself for example) was calculated. If there were missing values in all three replicates of the 0 µM Cu(II) growth condition, then the lowest abundance value identified in the entire dataset was used in its place (here the value 62658 was used). For fold changes between 2 and −2, a value of 0 was put in that data field to more easily visualize the fold changes in the heat map.

### Heat Maps of Changing Protein Abundances

1851 proteins were identified after the protein rollup. The z-score of the abundance value was calculated. This z-score normalization effectively adjusts all the protein abundances to the same scale to facilitate visualization of the abundance changes relative to an average or defined central value. This z-score uses the protein abundance value subtracted by the median value of the 0 µM Cu(II) growth condition for the 16, 22, or 32 hr dataset divided by the standard deviation for the 16, 22, or 32 hr dataset. These z-score values obtained for the 16, 22 and 32 hr datasets were combined into one text file table, were sorted based on the presence of the protein in all runs multiplied by the sum of the z-score and this order of z-score values was visualized in MeV (http://www.tm4.org). The fold changes calculated in the previous section were converted into a text file and imported into MeV (http://www.tm4.org).

## Supporting Information

Table S1 Median response of oxidative stress proteins in K. radiotolerans cultures during onset (16 hr) and mid (22 hr) exponential and stationary (32 hr) growth phases at varying concentrations of Cu(II). Response changes in protein abundance were calculated for all copper treatments relative to the no copper controls. The number of peptides detected for each protein is provided in parentheses.
(0.03 MB DOC)

Table S2 Median response of proteins involved in replication, repair, and recombination in K. radiotolerans during onset (16 hr) and mid (22 hr) exponential and stationary (32 hr) growth phases at varying concentrations of Cu(II). Response changes in protein abundance were calculated for all copper treatments relative to the no copper controls. The number of peptides detected for each protein is provided in parentheses.
(0.04 MB DOC)

Table S3 Median response of detoxification and defense proteins in K. radiotolerans cultures during onset (16 hr) and mid (22 hr) exponential and stationary (32 hr) growth phases at varying concentrations of Cu(II). Response changes in protein abundance were calculated for all copper treatments relative to the no copper controls. The number of peptides detected for each protein is provided in parentheses.
(0.03 MB DOC)

Table S4 Median response of membrane transport proteins in K. radiotolerans during onset (16 hr) and mid (22 hr) exponential and stationary (32 hr) growth phases at varying concentrations of Cu(II). Response changes in protein abundance were calculated for all copper treatments relative to the no copper controls. The number of peptides detected for each protein is provided in parentheses.
(0.04 MB DOC)

Table S5 Median response of protein regulators in K. radiotolerans during onset (16 hr) and mid (22 hr) exponential and stationary (32 hr) growth phases at varying concentrations of Cu(II). Response changes in protein abundance were calculated for all copper treatments relative to the no copper controls. The number of peptides detected for each protein is provided in parentheses.
(0.04 MB DOC)

Table S6 Median response of ribosomal proteins in K. radiotolerans during onset (16 hr) and mid (22 hr) exponential and stationary (32 hr) growth phases at varying concentrations of Cu(II). Response changes in protein abundance were calculated for all copper treatments relative to the no copper controls. The number of peptides detected for each protein is provided in parentheses.
(0.04 MB DOC)

Table S7 Median response of proteins involved in lipid metabolism in K. radiotolerans during onset (16 hr) and mid (22 hr) exponential and stationary (32 hr) growth phases at varying concentrations of Cu(II). Response changes in protein abundance were calculated for all copper treatments relative to the no copper controls. The number of peptides detected for each protein is provided in parentheses.
(0.04 MB DOC)

## References

[1] Phillips RW, Wiegel J, Berry CJ, Fliermans C, Peacock AD (2002). *Kineococcus radiotolerans* sp. nov., a radiation-resistant, Gram-positive bacterium.. Int J Syst Evol Microbiol.

[2] Radajewski S, Duxbury T (2001). Motility responses and desiccation survival of zoospores from the actinomycete *Kineosporia* sp. strain SR11.. Microb Ecol.

[3] Tringe SG, von Mering C, Kobayashi A, Salamov AA, Chen K (2005). Comparative metagenomics of microbial communities.. Science.

[4] Garrity GM, Heimbuch BK, Gagliardi M (1996). Isolation of zoosporogenous actinomycetes from desert soils.. Ind Microbiol Biotechnol.

[5] Schabereiter-Gurtner C, Piñar G, Vybiral D, Lubitz W, Rolleke S (2001). Rubrobacter-related bacteria associated with rosy discolouration of masonry and lime wall paintings.. Arch Microbiol.

[6] Torre de la JR, Goebel BM, Friedmann EI, Pace NR (2003). Microbial diversity of cryptoendolithic communities from the McMurdo Dry Valleys, Antarctica.. Appl Environ Microbiol.

[7] Kim TK, Garson MJ, Fuerst JA (2005). Marine actinomycetes related to the ‘Salinospora’ group from the Great Barrier Reef sponge *Pseudoceratina clavata.*. Environ Microbiol.

[8] Mincer TJ, Jensen PR, Kauffman CA, Fenical W (2002). Widespread and persistent populations of a major new marine actinomycete taxon in ocean sediments.. Appl Environ Microbiol.

[9] Wohl DL, McArthur JV (1998). Actinomycetes-flora associated with submersed freshwater macrophytes.. FEMS Microbiol Ecol.

[10] Bagwell CE, Milliken CE, Ghoshroy S, Blom DA (2008). Intracellular copper accumulation enhances the growth of *Kineococcus radiotolerans* during chronic irradiation.. Appl Environ Microbiol.

[11] Lee SD (2006). *Kineococcus marinus* sp. nov., isolated from marine sediments of the coast of Jeju, Korea.. Int J Syst Evol Microbiol.

[12] Yokota A, Tamura T, Nishii T, Hasegawa T (1993). *Kineococcus aurantiacus* gen. nov., sp. nov., a new aerobic, gram-positive, motile coccus with meso-diaminopimelic acid and arabinogalactan in the cell wall.. Int J Syst Bacteriol.

[13] O'Halloran TV, Culotta VC (2000). Metallochaperones, an intracellular shuttle service for metal ions.. J Biol Chem.

[14] Rae TD, Schmidt PJ, Pufahl RA, Culotta VC, O'Halloran TV (1999). Undetectable intracellular free copper: the requirement of a copper chaperone for superoxide dismutase.. Science.

[15] Lu ZH, Dameron CT, Solioz M (2003). The *Enterococcus hirae* paradigm of copper homeostasis: Copper chaperone turnover, interactions, and transactions.. BioMetals.

[16] Silver S, Phung LT (1996). Bacterial heavy metal resistance: New surprises.. Ann Rev Microbiol.

[17] Cooksey DA (1993). Copper uptake and resistance in bacteria.. Mol Microbiol.

[18] Odermatt A, Krapf R, Solioz M (1994). Induction of the putative copper ATPases, CopA and CopB, of *Enterococcus hirae* by Ag^+^ and Cu^2+^, and Ag^+^ extrusion by CopB.. Biochem Biophys Res Commun.

[19] Lu ZH, Solioz M (2001). Copper induced proteolysis of the CopZ copper chaperone of *Enterococcus hirae.*. J Biol Chem.

[20] Cha J, Cooksey DA (1991). Copper resistance in *Pseuodomonas syringae* mediated by periplasmic and outer membrane proteins.. Proc Natl Acad Sci USA.

[21] Cooksey DA, Azad HR (1992). Accumulation of copper and other metals by copper-resistant plant-pathogenic and saprophytic pseudomonads.. Appl Environ Microbiol.

[22] Macomber L, Rensing C, Imlay JA (2007). Intracellular copper does not catalyze the formation of oxidative DNA damage in *Escherichia coli.*. J Bacteriol.

[23] Coombs JT, Franco CMM (2003). Isolation and identification of actinobacteria from surface-sterilized wheat roots.. Appl Environ Microbiol.

[24] Gómez-Silva B, Rainey FA, Warren-Rhodes KA, McKay CP, Navarro-González R, Dion P, Nautiyal CS (2008). Atacama desert soil microbiology.. Microbiology of Extreme Soils, Soil Biology, Vol 13.

[25] Kuhlman KR, Fusco WG, La Duc MT, Allenbach LB, Ball CL (2006). Diversity of microorganisms within rock varnish in the Whipple Mountains, California.. Appl Environ Microbiol.

[26] West TS, Coombs TL (1981). Soil as the source of trace elements. Philosophical Transactions of the Royal Society of London. Series B.. Biological Sciences.

[27] Imperi F, Caneva G, Cancellieri L, Ricci MA, Sodo A (2007). The bacterial aetiology of rosy discoloration of ancient wall paintings.. Environ Microbiol.

[28] Urzí C, Brusetti L, Salamone P, Sorlini C, Stackebrandt E (2001). Biodiversity of Geodermatophilaceae isolated from altered stones and monuments in the Mediterranean basin.. Environ Microbiol.

[29] Urzí C, Salamone P, Schumann P, Stackebrandt E (2000). *Marmoricola aurantiacus* gen. nov., sp. nov., a coccoid member of the family *Nocardioidaceae* isolated from a marble statue.. Int J Syst Evol Microbiol.

[30] Albarracín VH, Amoroso MJ, Abate CM (2005). Isolation and characterization of indigenous copper-resistant actinomycete strains.. Chemie der Erde – Geochemistry.

[31] Fredrickson JK, Zachara JM, Balkwill DL, Kennedy D, Li SMW (2004). Geomicrobiology of high-level nuclear waste-contaminated vadose sediments at the Hanford site, Washington State.. Appl Environ Microbiol.

[32] Gremion F, Chatzinotas A, Harms H (2003). Comparative 16S rDNA and 16S rRNA sequence analysis indicates that *Actinobacteria* might be a dominant part of the metabolically active bacteria in heavy metal-contaminated bulk and rhizosphere soil.. Environ Microbiol.

[33] Haferburg G, Merten D, Büchel G, Kothe E (2007). Biosorption of metal and salt tolerant microbial isolates from a former uranium mining area. Their impact on changes in rare earth element patterns in acid mine drainage.. J Bas Microbiol.

[34] Konstantinidis KT, Isaacs N, Fett J, Simpson S, Long DT (2003). Microbial diversity and resistance to copper in metal-contaminated lake sediment.. Microb Ecol.

[35] Schmidt A, Haferburg G, Sineriz M, Merten D, Büchel G (2005). Heavy metal resistance mechanisms in actinobacteria for survival in AMD contaminated soils.. Chemie der Erde – Geochem.

[36] Van Nostrand JD, Khijniak TV, Gentry TJ, Novak MT, Sowder AG (2007). Isolation and characterization of four Gram-positive nickel-tolerant microorganisms from contaminated sediments.. Micro Ecol.

[37] Ishiguro EE, Wolfe RS (1970). Control of morphogenesis in *Geodermatophilus*: Ultrastructural studies.. J Bacteriol.

[38] Luedemann GM (1968). *Geodermatophilus,* a new genus of the *Dermatophilaceae* (*Actinomycetales*).. J Bacteriol.

[39] Williams ST, Sharpe ME, Holt JG (1989). Bergey's manual of systematic bacteriology, Vol 4.. Williams & Wilkins.

[40] Johnston DW, Cross T (1976). Actinomycetes in lake muds: dormant spores or metabolically active mycelium?. Freshwater Biol.

[41] Mayfield CI, Williams ST, Ruddick SM, Hatfield HL (1972). Studies on the ecology of actinomycetes in soil IV. Observations on the form and growth of streptomycetes in soil.. Soil Biol Biochem.

[42] Baikun L, Bishop PL (2004). Micro-profiles of activated sludge floc determined using microelectrodes.. Water Res.

[43] Daly MJ, Gaidamakova EK, Matrosova VY, Vasilenko A, Zhai M (2004). Accumulation of Mn(II) in *Deinococcus radiodurans* facilitates gamma-radiation resistance.. Science.

[44] Silva JC, Gorenstein MV, Li GZ, Vissers JP, Geromanos SJ (2006). Absolute quantification of proteins by LCMSE: a virtue of parallel MS acquisition.. Mol Cell Proteomics.

[45] Gupta N, Pevzner PA (2009). False discovery of protein identifications: a strike against the two-peptide rule.. J Proteome Res.

[46] Kim S, Gupta N, Pevzner PA (2008). Spectral probabilities and generating functions of tandem mass spectra: a strike against decoy databases.. J Proteome Res.

[47] Bagwell CE, Bhat S, Hawkins GM, Smith BW, Biswas T (2008). Survival in nuclear waste, extreme resistance, and potential applications gleaned from the genome sequence of *Kineococcus radiotolerans* SRS30216.. PLoS One.

[48] Ellington MJK, Sawers G, Sears HJ, Spiro S, Richardson DJ (2003). Characterization of the expression and activity of the periplasmic nitrate reductase of *Paracoccus pantotrophus* in chemostat cultures.. Microbiol.

[49] Ansaldi M, Théraulaz L, Baraquet C, Panis G, Méjean V (2007). Aerobic TMAO respiration in *Escherichia coli.*. Mol Microbiol.

[50] Deaconescu AM, Savery N, Darst SA (2007). The bacterial transcription-repair coupling factor.. Curr Opin Struct Biol.

[51] Qing Z, Xinjue Z, Hong XU, Bujin XU, Yuejin H (2006). RadA: A protein involved in DNA damage repair processes of *Deinococcus radiodurans* R1.. Chinese Sci Bull.

[52] Song Y, Sargentini NJ (1996). *Escherichia coli* DNA repair genes *radA* and *sms* are the same gene.. J Bacteriol.

[53] Su M, Cavallo S, Stefanini S, Chiancone E, Chasteen ND (2005). The so-called *Listeria innocua* ferritin is a Dps protein. Iron incorporation, detoxification, and DNA protection properties.. Biochem.

[54] Dameron CT, Harrison MD (1998). Mechanisms for protection against copper toxicity.. Am J Clin Nutr.

[55] Denisov ET, Afanas'ev IB, Denisov ET, Afanas'ev IB (2005). DNA oxidative damage.. Oxidation and Antioxidants in Organic Chemistry and Biology.

[56] Erardi FX, Failla ML, Falkinham JO (1987). Plasmid-encoded copper resistance and precipitation by *Mycobacterium scrofulaceum.*. Appl Environ Microbiol.

[57] Fabisiak JP, Tyurin VA, Tyurina YY, Borisenko GG, Korotaeva A (1999). Redox regulation of copper-metallothionein.. Arch Biochem Biophy.

[58] Keyer K, Imlay JA (1996). Superoxide accelerates DNA damage by elevating free-iron levels.. Proc Natl Acad Sci USA.

[59] Hassan HM, Fridovich I (1977). Regulation of the synthesis of superoxide dismutase in *Escherichia coli.*. J Biol Chem.

[60] Culotta VC, Joh HD, Lin SJ, Slekar KH, Strain J (1995). A physiological role for *Saccharomyces cerevisiae* copper/zinc superoxide dismutase in copper buffering.. J Biol Chem.

[61] Geslin C, Llanos J, Prieur D, Jeanthon C (2001). The manganese and iron superoxide dismutases protect *Escherichia coli* from heavy metal toxicity.. Res Microbiol.

[62] Fridovich I (1983). Superoxide radical: an endogenous toxicant.. Ann Rev Pharmacol Toxicol.

[63] Scott MD, Meshnick SR, Eaton JW (1987). Superoxide dismutase-rich bacteria.. J Biol Chem.

[64] Schwartz CE, Krall J, Norton L, McKay K, Kay D (1983). Catalase and superoxide dismutase in *Escheriichia coli.*. J Biol Chem.

[65] Loewen PC (1984). Isolation of catalase-deficient *Escherichia coli* mutants and genetic mapping of *katE,* a locus that affects catalase activity.. J Bacteriol.

[66] Hassan HM, Fridovich I (1979). Intracellular production of superoxide radical and hydrogen peroxide by redox active compounds.. Arch Biochem Biophys.

[67] Kelner MJ, Bagnell R (1990). Alteration of endogenous glutathione peroxidase, manganese superoxide dismutase, and glutathione transferase activity in cells transfected with a copper-zinc superoxide dismutase expression vector.. J Biol Chem.

[68] Rosadini CV, Wong SMS, Akerley BJ (2008). The periplasmic disulfide oxidoreductase DsbA contributes to *Haemophilus influenzae* pathogenesis.. Infect and Immunity.

[69] Peek JA, Taylor RK (1992). Characterization of a periplasmic thiol:disulfide interchange protein required for the functional maturation of secreted virulence factors of *Vibrio cholerae.*. Proc Natl Acad Sci USA.

[70] Bringer MA, Rolhion N, Glasser AL, Darfeuille-Michaud A (2007). The oxidoreductase DsbA plays a key role in the ability of the Crohn's disease-associated adherent-invasive *Escherichia coli* strain LF82 to resist macrophage killing.. J Bacteriol.

[71] Swaving J, De Bont JAM, Westphal A, De Kok A (1996). A novel type pyridine nucleotide-disulfide oxidoreductase is essential for NAD^+^- and NADPH-dependent degradation of epoxyalkanes by *Xanthobacter* strain Py2.. J Bacteriol.

[72] Attfield PV (1987). Trehalose accumulates in *Saccharomyces cerevisiae* during exposure to agents that induce heat shock response.. FEBS Letters.

[73] De Smet KAL, Weston A, Brown IN, Young DB, Robertson BD (2000). Three pathways for trehalose biosynthesis in mycobacteria.. Microbiol.

[74] Monobe M, Uzawa A, Hino M, Ando K, Kojima S (2005). Glycine betaine, a beer component, protects radiation-induced injury.. J Radiat Res.

[75] Grassmann J, Hippeli S, Elstner EF (2002). Plant's defence and its benefits for animals and medicine: role of phenolics and terpenoids in avoiding oxygen stress.. Plant Physiol Biochem.

[76] De Mot R, Nagy I, Walz J, Baumeister W (1999). Proteasomes and other self-compartmentalizing proteases in prokaryotes.. Trends in Microbiol.

[77] Liu M, Peng F, Wang Y, Zhang K, Chen G (2009). *Kineococcus xinjiangensis* sp. nov., isolated from desert sand.. Int J Syst Evol Microbiol.

[78] Han WD, Kawamoto S, Hosoya Y, Fujita M, Sadaie Y (1998). A novel sporulation-control gene (spoOM) of *Bacillus subtilis* with a sigma-H-regulated promoter.. Gene.

[79] Fuqua WC, Winans SC, Greenberg EP (1994). Quorum sensing in bacteria: the LuxR-LuxI family of cell density-responsive transcriptional regulators.. J Bacteriol.

[80] Achard-Joris M, Moreau JL, Lucas M, Baudrimont M, Mesmer-Dudons N (2007). Role of metallothioneins in superoxide radical generation during copper redox cycling: Defining the fundamental function of metallothioneins.. Biochimie.

[81] Kodali VP, Sen R (2008). Antioxidant and free radical scavenging activities of an exopolysaccharide from a probiotic bacterium.. Biotechnol J.

[82] Milagres AMF, Muchuca A, Napoleão D (1999). Detection of siderophore production from several fungi and bacteria by a modification of chrome azurol S (CAS) agar plate assay.. J Microbiol Methods.

[83] Makarova KS, Omelchenko MV, Gaidamokova EK, Matrosova VY, Vasilenko A (2007). *Deinococcus geothermalis:* The pool of extreme radiation resistance genes shrinks.. PLoS ONE.

[84] Masselon C, Pasa-Tolic L, Tolic N, Anderson GA, Bogdanov B (2005). Targeted comparative proteomics by liquid chromatography-tandem fourier ion cyclotron resonance mass spectrometry.. Anal Chem.

[85] Qian WJ, Liu T, Monroe ME, Strittmatter EF, Jacobs JM (2005). Probability-based evaluation of peptide and protein identifications from tandem mass spectrometry and SEQUEST analysis: The human proteome.. J Proteome Res.

[86] Shen Y, Tolic N, Zhao R, Pasa-Tolic L, Li L (2001). High-throughput proteomics using high-efficiency multiple-capillary liquid chromatography with on-line high-performance ESI FTICR mass spectrometry.. Anal Chem.

[87] Smith RD, Anderson GA, Lipton MS, Pasa-Tolic L, Shen Y (2002). An accurate mass tag strategy for quantitative and high-throughput proteome measurements.. Proteomics.

[88] Eng JK, McCormack AL, Yates JR (1994). An approach to correlate tandem mass spectral data of peptides with amino acid sequences in a protein database.. J Am Soc Mass Spectrom.

[89] Lopez-Ferrer D, Martinez-Bartolome S, Villar M, Campillos M, Martin-Maroto F (2004). Statistical model for large-scale peptide identification in databases from tandem mass spectra using SEQUEST.. Anal Chem.

[90] Zimmer JS, Monroe ME, Qian WJ, Smith rd (2006). Advances in proteomics data analysis and display using an accurate mass and time tag approach.. Mass Spectrom Rev.

[91] Polpitiya AD, Qian WJ, Jaitly N, Petyuk VA, Adkins JN (2008). DAnTE: a statistical tool for quantitative analysis of –omics data.. Bioinformatics.

