# Supplementary material for: Proteomic and Physiological Responses of Kineococcus radiotolerans to Copper
Source: PLoS One. 2010 Aug 26;5(8):e12427. doi: 10.1371/journal.pone.0012427 (PMC2928746; doi:10.1371/journal.pone.0012427)
Supplement: Table S1 — Median response of oxidative stress proteins in K. radiotolerans cultures during onset (16 hr) and mid (22 hr) exponential and stationary (32 hr) growth phases at varying concentrations of Cu(II). Response changes in protein abundance were calculated for all copper treatments relative to the no copper controls. The number of peptides detected for each protein is provided in parentheses. (0.03 MB DOC) [file pone.0012427.s001.doc]

**Table S1.** Median response of oxidative stress proteins in *K. radiotolerans* cultures during onset (16 hr) and mid (22 hr) exponential and stationary (32 hr) growth phases at varying concentrations of Cu(II). Response changes in protein abundance were calculated for all copper treatments relative to the no copper controls. The number of peptides detected for each protein is provided in parentheses.

| **16hr 22hr 32hr**  **Locus Protein** 0.1mM 0.75mM 1.5mM 0.1mM 0.75mM 1.5mM 0.1mM 0.75mM 1.5mM |
| --- |
| Krad0128 Chloride peroxidase (2) 2.39 3.83 - 2.37 - 4.39 - 4.21 11.52  Krad0152 NADH:flavin oxidoreductase/NADH oxidase (5) - 2.40 3.79 - 2.11 3.93 - 3.43 -  Krad0759 Pyridoxamine 5'-phosphate oxidase-related FMN-binding (4) - 2.58 3.92 - - 2.16 - 5.65 6.29  Krad0815 Catalase (5) - - - - - - -6.34 -3.76 -  Krad0838 Putrescine oxidase (13) - - - - -3.94 - - - -  Krad0848 Thioredoxin (7) - 2.64 2.92 3.44 3.67 5.63 -2.08 - 3.91  Krad1001 FAD linked oxidase domain protein (37) - - - - - - - 2.06 2.19  Krad1091 peptide methionine sulfoxide reductase MsrA (2) - - - - 3.00 2.85 - - 2.31  Krad1239 ABC transporter related (17) - 2.47 3.13 - - 2.41 - 3.50 2.95  Krad1586 protoporphyrinogen oxidase (8) - - 5.68 - - - - 2.28 6.50  Krad1804 pyridoxamine 5'-phosphate oxidase-relatedFMN-binding (4) - - - - - - - - 2.05  Krad2042 pyridoxamine 5'-phosphate oxidase-relatedFMN-binding (5) - - - - - - - 2.04 -  Krad2472 amine oxidase (1) - 2.02 2.83 - - 2.58 32.15 223.65 214.57  Krad2670 periplasmic binding protein (6) 2.06 3.71 5.07 2.40 - 2.68 - 2.26 3.08  Krad3258 Cytochrome c oxidase subunit I type (1) - -2.62 -11.66 - -3.85 -3.44 - - -  Krad3268 Dihydroorotate oxidase (9) - - - - - - - 2.18 2.63  Krad3350 Dyp-type peroxidase family (11) - - - - 2.05 - - 2.83 -  Krad3411 putative oxygen-independent coproporphyrinogenIII oxidase (2) - - 2.54 - - -2.61 - - 2.49  Krad3558 L-aspartate oxidase (1) - - 2.97 2.28 - 3.43 - - 4.04  Krad3704 transport system permease protein (1) - - - - - - - 5.74 6.57  Krad3713 pyridoxamine 5'-phosphate oxidase-relatedFMN-binding (1) - - 3.77 - - - - - -  Krad3757 alkyl hydroperoxide reductase Thiol specificantioxidant (7) - - - - - - 2.58 6.29 5.99  Krad3775 NADH:flavin oxidoreductase/NADH oxidase (3) - - - - - 2.03 - - 2.53  Krad3959 Formaldehyde dehydrogenase, glutathione-independent (23) - - 24.91 - 94.08 123.68 -12.67 -2.08 4.26  Krad4105 Siderophore-interacting protein (13) - 2.44 2.33 - - 3.25 - - -  Krad4106 FAD-binding 9 siderophore-interacting domain protein (12) - 2.13 2.46 - - 2.61 - 3.46 3.56  Krad4137 ABC transporter related (1) - 2.23 2.88 - - - - - -  Krad4160 FAD linked oxidase domain protein (17) - - 2.09 - - - - 2.03 - |
