# Supplementary material for: Proteomic and Physiological Responses of Kineococcus radiotolerans to Copper
Source: PLoS One. 2010 Aug 26;5(8):e12427. doi: 10.1371/journal.pone.0012427 (PMC2928746; doi:10.1371/journal.pone.0012427)
Supplement: Table S2 — Median response of proteins involved in replication, repair, and recombination in K. radiotolerans during onset (16 hr) and mid (22 hr) exponential and stationary (32 hr) growth phases at varying concentrations of Cu(II). Response changes in protein abundance were calculated for all copper treatments relative to the no copper controls. The number of peptides detected for each protein is provided in parentheses. (0.04 MB DOC) [file pone.0012427.s002.doc]

**Table S2.** *.* Median response of proteins involved in replication, repair, and recombination in *K. radiotolerans* during onset (16 hr) and mid (22 hr) exponential and stationary (32 hr) growth phases at varying concentrations of Cu(II). Response changes in protein abundance were calculated for all copper treatments relative to the no copper controls. The number of peptides detected for each protein is provided in parentheses.

| **16hr 22hr 32hr**  **Locus Protein** 0.1mM 0.75mM 1.5mM 0.1mM 0.75mM 1.5mM 0.1mM 0.75mM 1.5mM |
| --- |
| Krad4243 Dcd, dCTP deaminase (5) - - - - - - - - 2.48  Krad0001 DnaA, Chromosomal replication initiator protein (19) - - - - - 2.14 - -2.35 -  Krad4333 DnaB, Replicative DNA helicase (7) - - 2.75 - - - - 6.17 5.55  Krad3361 DnaE, DNA polymerase III (haloenzyme) α subunit (10) - - - - - - - 3.08 4.19  Krad0002 DnaN, DNA polymerase III (haloenzyme) β subunit (18) - - - - - - - - 5.90  Krad4503 DnaQ, DNA polymerase III (haloenzyme) ε subunit (14) - - - - 2.43 - - - -  Krad0466 DnaZ/X, DNA polymerase III (haloenzyme) γ/τ subunit (5) - - - - - - - 2.18 2.63  Krad1557 Dut, dUTPase (6) - - - - - - - - 2.06  Krad3612 ERCC3, XPB/ERCC3 helicase (6) - - - - - - - 2.08 -  Krad2983 Fmu, rRNA SAM-dependent methyltransferase (15) - - 2.31 - - - - 2.45 3.06  Krad0007 GyrA, DNA gyrase subunit A (20) - - - - - - - 2.08 -  Krad3762 HAM1/YggV, Xantosine triphosphate pyrophosphatase (6) - - 4.78 2.59 2.89 - - 4.81 5.19  Krad0173 HelY, Probable helicase, Ski2 subfamily (3) - 3.30 - - - - - - -  Krad1360 HupB/IHF, DNA binding protein II, Integration host factor (5) - - - - - 2.19 - -3.38 -  Krad2005 HupB/IHF, DNA binding protein II, Integration host factor (5) - - - - - - -3.88 -2.81 -  Krad2805 HupB/IHF, DNA binding protein II, Integration host factor (5) - - - - - - -2.07 - -  Krad3371 HupB/IHF, DNA binding protein II, Integration host factor (10) - - - - 4.72 2.41 2.23 -3.11 -3.18  Krad0653 LigC, Probable DNA ligase (6) - - 2.17 - - - - - -  Krad1067 Mfd, Transcription repair coupling factor, helicase (13) - - - - 2.34 - - 2.89 2.25  Krad3140 MutT, 8-oxo-dGTPase (12) - - - - - - - 9.78 11.23  Krad1546 ParC, DNA topoisomerase IV, subunit A (13) - - - - - - - - 4.82  Krad1534 ParE, DNA topoisomerase IV, subunit B (16) - - - - - - - 2.46 3.10  Krad3276 PepA, DNA binding protein (28) - - - - 3.89 5.11 13.12 34.47 -  Krad1149 PepA, DNA binding protein (21) - - 2.21 - 2.28 4.06 7.45 16.66 21.81  Krad2988 PriA, Putative primosomal protein n′ (replication factor Y) (2) - - - - - - - - 2.03  Krad1492 RecA, Recombinase, ssDNA-dependent ATPase, LexA activator (28) - - 2.17 - - 2.03 - 2.16 3.08  Krad0004 RecF, Predicted ATPase, daughter-strand gap repair (7) - - 2.12 - - - - - 3.43  Krad1368 RecG, Holliday junction specific DNA helicase (9) - - - - 2.45 - - 2.86 2.81  Krad3147 RecN, Predicted ATPase (6) - - - - - - - 2.47 2.20  Krad4305 RecQ, Helicase, suppressor of illegitimate recombination (5) - - 5.56 2.75 -2.57 -6.29 -8.52 -2.99 -2.86  Krad0465 RecR, Daughter strand gap repair (6) - - - - - - - 2.25 3.89  Krad2553 SbcC, ss endonuclease 3’-5’ ds exonuclease (5) - - - - - 3.20 3.57 9.08 3.52  Krad3772 SdrA, DNA or RNA helicase superfamily II (4) - - 2.17 - - 3.46 4.44 - -  Krad1242 SrmB, Superfamily II ATP-dependent helicase (15) - - 2.21 - - - - 2.90 4.75  Krad0858 SrmB, Superfamily II ATP-dependent helicase (13) - - - - 4.10 2.86 2.95 6.00 8.68  Krad4338 Ssb, Single strand binding protein (7) - - - - - - -2.19 -2.04 -  Krad3900 UDP-Glucose 6-dehydrogenase (52) - - - - - - - - 3.00  Krad3639 Ung, Uracil DNA glycosylase (7) - - - - - - - - 2.16  Krad2942 Excinuclease ABC subunit B (5) - - - - - - - - 2.70  Krad1179 UvrD, DNA helicase II, initiates unwinding from a nick (8) - 2.03 - - - - - - -  Krad1121 XseB, Exonuclease VII, small subunit (4) - - 2.01 - 2.24 - - - 2.21  Krad3979 XthA, Exodeoxyribonuclease III (AP endonuclease) (11) - - - - - - - - 2.07  Krad3612 YejH, DNA or RNA helicase superfamily II (6) - - - - - - - 2.08 - |
