# Supplementary material for: Proteomic and Physiological Responses of Kineococcus radiotolerans to Copper
Source: PLoS One. 2010 Aug 26;5(8):e12427. doi: 10.1371/journal.pone.0012427 (PMC2928746; doi:10.1371/journal.pone.0012427)
Supplement: Table S3 — Median response of detoxification and defense proteins in K. radiotolerans cultures during onset (16 hr) and mid (22 hr) exponential and stationary (32 hr) growth phases at varying concentrations of Cu(II). Response changes in protein abundance were calculated for all copper treatments relative to the no copper controls. The number of peptides detected for each protein is provided in parentheses. (0.03 MB DOC) [file pone.0012427.s003.doc]

**Table S3.** Median response of detoxification and defense proteins in *K. radiotolerans* cultures during onset (16 hr) and mid (22 hr) exponential and stationary (32 hr) growth phases at varying concentrations of Cu(II). Response changes in protein abundance were calculated for all copper treatments relative to the no copper controls. The number of peptides detected for each protein is provided in parentheses.

| **16hr 22hr 32hr**  **Locus Protein** 0.1mM 0.75mM 1.5mM 0.1mM 0.75mM 1.5mM 0.1mM 0.75mM 1.5mM |
| --- |
| Krad0128 Chloride peroxidase (2) 2.39 3.83 - 2.37 - 4.39 - 4.21 11.52  Krad3757 Alkyl hydroperoxide reductase, AhpC (7) - - - - - - 2.58 6.29 5.99  Krad0815 Mn – Catalase, KatA (5) - - - - - - -6.34 -3.76 -  Krad0848 Thioredoxin (7) - 2.64 2.92 3.44 3.67 5.63 -2.08 - 3.91  Krad1091 Peptide methionine sulfoxide reductase MsrA (2) - - - - 3.00 2.85 - - 2.31  Krad3350 Dyp-type peroxidase (11) - - - - 2.05 - - 2.83 -  Krad0398 Putative vancomycin resistance protein, VanW (2) - - - - - - - 2.30 2.28  Krad0860 Putative β-lactamase binding protein (4) - 2.38 11.47 4.23 5.13 - - 3.42 5.03  Krad0951 Putative β-lactamase binding protein (16) - - - 2.94 - - 2.19 - -3.34  Krad1116 ABC-type antimicrobial peptide transport ATPase (9) - - 2.42 - - - - - 2.20  Krad1284 Putative β-lactamase binding protein (14) - - 2.43 - - 2.63 2.66 - 3.92  Krad1868 Multidrug resistance efflux pump (4) - - - - - - - - 2.44  Krad2553 ABC-type antimicrobial peptide transport ATPase (5) - - - - - 3.20 3.57 9.08 3.52  Krad2915 ABC-type antimicrobial peptide transport ATPase (23) - - 2.33 - - - - 3.50 2.95  Krad3287 Putative β-lactamase binding protein (12) - - 3.82 2.37 2.55 2.48 2.37 6.16 5.26  Krad3551 Restriction endonuclease (1) - - -8.41 - - - - - -  Krad3705 ABC-type antimicrobial peptide transport ATPase (7) - - - - 2.61 2.61 5.77 5.19 -  Krad3228 CrtL, Phytoene desaturase (14) - - 2.12 - - 2.91 2.99 5.96 11.15 Krad3229 CrtB, Phytoene synthase (10) - - 2.37 - - - - - - |
