# Supplementary material for: Proteomic and Physiological Responses of Kineococcus radiotolerans to Copper
Source: PLoS One. 2010 Aug 26;5(8):e12427. doi: 10.1371/journal.pone.0012427 (PMC2928746; doi:10.1371/journal.pone.0012427)
Supplement: Table S4 — Median response of membrane transport proteins in K. radiotolerans during onset (16 hr) and mid (22 hr) exponential and stationary (32 hr) growth phases at varying concentrations of Cu(II). Response changes in protein abundance were calculated for all copper treatments relative to the no copper controls. The number of peptides detected for each protein is provided in parentheses. (0.04 MB DOC) [file pone.0012427.s004.doc]

**Table S4.**  Median response of membrane transport proteins in *K. radiotolerans* during onset (16 hr) and mid (22 hr) exponential and stationary (32 hr) growth phases at varying concentrations of Cu(II). Response changes in protein abundance were calculated for all copper treatments relative to the no copper controls. The number of peptides detected for each protein is provided in parentheses.

| **16hr 22hr 32hr**  **Locus Protein** 0.1mM 0.75mM 1.5mM 0.1mM 0.75mM 1.5mM 0.1mM 0.75mM 1.5mM |
| --- |
| Krad0057 ABC transporter related (7) - - - 2.05 - - 2.01 2.50 4.83  Krad0396 ABC transporter ATP-binding protein (3) - - - - - - - - 2.37  Krad0440 ABC transporter related (2) - - - -4.29 -14.18 - 8.15 2.71 -  Krad0452 ABC transporter related (11) - - - - - - - - 3.63  Krad0546 ABC transporter related (1) - - 3.63 - - 7.13 - - -  Krad0579 ABC transporter related (32) - - 2.52 - 2.70 2.53 - - 3.50  Krad0580 Putative ABC transporter transmembrane protein (1) 3.41 2.61 4.28 2.86 2.34 3.55 - - 3.39  Krad0748 ABC transporter transmembrane region (1) - -2.15 -3.17 - - - - - -  Krad0783 Heavy metal transport/detoxification protein (3) - 4.46 20.38 - 4.82 7.47 4.04 14.58 39.13  Krad0872 Phosphate ABC transporter, ATPase subunit (11) - - - - - - - 2.27 2.31  Krad0875 Phosphate ABC transporter, periplasmic (15) - - - - - - - 2.12 -  Krad0916 ABC transporter related (30) - - - - - - - - 2.19  Krad1041 Conserved hypothetical protein (4) - - 2.45 - - - -2.51 - -2.15  Krad1047 ABC transporter related (9) - - - - - - - 2.52 2.70  Krad1084 ABC transporter related (3) - - - - - 2.20 - 2.47 2.64  Krad1116 ABC transporter related (9) - - 2.42 - - 2.35 -2.40 - 2.01  Krad1187 ABC transporter related (4) - - - - - - - - 2.08  Krad1239 ABC transporter related (17) - 2.47 3.13 - - 2.41 - 3.50 2.95  Krad1271 H+transporting two-sector ATPase delta/epsilonsubunit (3) - - - - 2.97 - - - -  Krad1290 ABC transporter related (3) - - 2.74 - - - - 2.28 -  Krad1293 Substrate-binding region, ABC-type glycinebetaine transporter (9) - - - - - - - - 2.38  Krad1459 ABC transporter related (8) - - - - - - - 2.00 2.39  Krad1498 ABC transporter related (10) - - - - - 2.05 - - -  Krad1563 Putative transporter (2) - - - 2.70 - 3.92 -2.05 - -  Krad1832 Mg2 transporter protein CorA family protein (1) - - - - - - - - 2.08  Krad2093 ABC transporter related (8) - - - 2.75 - - -2.06 - 4.49  Krad2094 ABC transporter related (9) - - - - - 2.35 - 2.78 5.04  Krad2122 ABC transporter related (2) - -2.55 3.56 - - - - -3.82 -  Krad2897 ABC transporter related (17) - - - - - - - - 2.28  Krad2910 ABC transporter related (5) - - - - - - - 2.66 2.80  Krad2919 ABC transporter related (10) - - - - - - - 5.09 2.29  Krad2953 ABC transporter related (3) - - - 2.65 3.66 - - 6.98 4.96  Krad3061 Conserved hypothetical protein (2) - - -5.31 - -2.17 - 4.44 - -  Krad3113 ABC transporter related (4) - - 2.14 - - 2.68 - 2.61 3.01  Krad3513 Drug resistance transporter, EmrB/QacAsubfamily (9) - 2.16 23.74 - 2.64 3.65 -10.49 -4.06 -5.65  Krad3531 MscS Mechanosensitive ion channel (4) - - - - - - - 2.88 2.15  Krad3652 ABC transporter related (2) -2.57 - - 2.34 - - -2.12 4.30 -  Krad3704 Transport system permease protein (1) - - - - - - - 5.74 6.57  Krad3705 ABC transporter related (7) - - - 2.12 - 2.56 - - 3.26  Krad3885 ABC transporter related (4) - - 2.44 - - - - - -  Krad3923 Daunorubicin resistance ABC transporter ATPase subunit (9) - - 2.58 - 2.87 3.33 -2.44 2.17 4.57  Krad3948 ABC transporter related (11) - - - - - 2.33 - 3.31 2.23  Krad3975 ABC transporter related (16) - 2.11 2.68 - - 2.87 - 2.43 2.58  Krad3977 ABC transporter related (15) - 2.43 3.73 - 2.24 3.64 - 2.73 2.56  Krad4052 ABC transporter related (8) - - - - 2.19 - - 2.25 2.72  Krad4137 ABC transporter related (1) - 2.23 2.88 - - - - - -  Krad4173 Putative ABC transporter (9) - - 2.32 - - - - - -  Krad4174 ABC transporter related (22) - - - - - - - - 2.60  Krad4360 ABC transporter related (1) - - - - - - - -3.38 -  Krad4570 Cation diffusion facilitator family transporter (1) - - 2.26 - - - - - 2.71 |
