# Supplementary material for: Proteomic and Physiological Responses of Kineococcus radiotolerans to Copper
Source: PLoS One. 2010 Aug 26;5(8):e12427. doi: 10.1371/journal.pone.0012427 (PMC2928746; doi:10.1371/journal.pone.0012427)
Supplement: Table S5 — Median response of protein regulators in K. radiotolerans during onset (16 hr) and mid (22 hr) exponential and stationary (32 hr) growth phases at varying concentrations of Cu(II). Response changes in protein abundance were calculated for all copper treatments relative to the no copper controls. The number of peptides detected for each protein is provided in parentheses. (0.04 MB DOC) [file pone.0012427.s005.doc]

**Table S5.** Median response of protein regulators in *K. radiotolerans* during onset (16 hr) and mid (22 hr) exponential and stationary (32 hr) growth phases at varying concentrations of Cu(II). Response changes in protein abundance were calculated for all copper treatments relative to the no copper controls. The number of peptides detected for each protein is provided in parentheses.

| **16hr 22hr 32hr**  **Locus Protein** 0.1mM 0.75mM 1.5mM 0.1mM 0.75mM 1.5mM 0.1mM 0.75mM 1.5mM |
| --- |
| Krad0506 Transcriptional regulator, XRE family (1) 2.46 - 2.61 - - - 98.14 - 54.65  Krad1186 Periplasmic binding protein/LacI transcriptional regulator (9) - - - - 344.86 476.09 - 43.25 6.90  Krad2707 Transcriptional regulator, TetR family (1) 2.22 3.51 4.20 134.38 33.75 78.35 - 9.78 11.26  Krad3093 Putative transcriptional regulator, MerR family (1) - - 2.44 2.59 - 2.47 - 11.37 2.11  Krad0198 Transcriptional regulator IclR (4) - 2.15 - - 2.50 - - 3.89 8.50  Krad3007 Transcriptional regulator, XRE family (6) - - - - - - 2.60 4.39 5.58  Krad1413 Transcriptional regulator, TraR/DksA family (2) - - - - - - 2.19 5.40 4.11  Krad2108 Two component transcriptional regulator, winged helix family (1) - - - - - - 3.32 4.94 3.87  Krad3930 Transcriptional regulator, MarR family (2) - - - - - - - 5.31 3.29  Krad1350 Two component transcriptional regulator, LuxR family (4) - - 2.21 - 4.62 2.43 - 2.06 4.68  Krad3528 Transcriptional regulator, winged helix family (3) - 7.15 - - 12.18 7.01 - 6.35 2.23  Krad3810 Two component transcriptional regulator, LuxR family (8) - - 2.09 - - - 3.34 3.60 4.03  Krad1879 Transcriptional regulator-like (5) - - - - - - - 2.90 4.20  Krad3694 Putative regulatory protein, FmdB family (5) 2.08 - 2.16 2.99 2.66 2.91 2.62 3.47 4.25  Krad1535 Transcriptional regulator, DeoR family (8) - - 2.11 - - - - 6.21 -  Krad3511 Transcriptional regulator, MarR family (3) - - - - - - - 2.99 3.77  Krad0913 Transcriptional regulator, MarR family (4) - - - - - - - 3.80 2.94  Krad4144 Putative transcriptional regulator, GntR family (1) - - - - - - - 2.68 3.67  Krad3437 Regulatory protein LuxR (1) - - - 2.06 - - - 2.98 2.84  Krad3370 Ferric uptake regulator, Fur family (3) 2.52 - - 2.53 - - 7.34 2.74 3.14  Krad4110 Putative transcriptional regulator, AsnC family (7) - - - 2.19 - 2.33 - 2.22 3.83  Krad3036 Two component transcriptional regulator, winged helix family (7) - 2.24 5.65 3.24 5.06 6.88 - - 4.82  Krad3121 Putative transcriptional regulator (4) - - - - - 2.02 - 3.63 -  Krad2043 Transcriptional regulator, MarR family (3) - 2.54 2.54 - - - - 2.48 2.55  Krad1921 Transcriptional regulator, GntR family (2) - - - - - - - 2.34 2.12  Krad2325 Regulatory protein LuxR (2) - - - - - - - - 3.00  Krad4054 Periplasmic binding protein/LacI transcriptional regulator (18) - - - - - - - - 3.63  Krad2729 Regulatory protein LuxR (2) 2.15 - - 2.62 - 3.15 - - 4.22  Krad0922 Regulatory protein LacI (8) 2.38 - - 2.50 - - 3.26 - 3.13  Krad2941 Transcriptional regulator, MarR family (4) - - - - - 3.46 - - 2.15  Krad2116 Transcriptional regulator, TetR family (1) - - 2.33 - - - - 2.00 2.14  Krad4318 Transcriptional regulator, TetR family (11) - - - - - - - - 2.93  Krad0424 Putative transcriptional regulator, Crp/Fnr family (2) - -2.03 - - - - - - 3.00  Krad0898 Transcriptional regulator, CarD family (12) - - - - - - 3.51 2.49 -  Krad2225 Transcriptional regulator IclR (1) - - - - - - - 2.07 -  Krad3815 Two component transcriptional regulator, winged helix family (6) - - - - - - - - 2.02  Krad2286 Regulatory protein LuxR (2) - - 2.93 - - 2.07 - - 2.96  Krad4251 Transcriptional regulator, MarR family (4) - - - - - - - - 2.32  Krad2681 Transcriptional regulator, GntR family (1) - - - - - - - 2.10 -  Krad2894 Transcriptional regulator, MarR family (5) - - 3.45 - - - - - -  Krad3595 Periplasmic binding protein/LacI transcriptional regulator (3) - - 2.93 2.06 3.19 2.78 - - -  Krad1230 Two component transcriptional regulator, winged helix family (18) - - 3.89 - - 2.45 - - -  Krad1457 Periplasmic binding protein/LacI transcriptional regulator (13) - - - - - - -2.51 - -  Krad4211 Transcriptional regulator, GntR family (1) - - 3.26 - -2.34 - - - -  Krad3728 Periplasmic binding protein/LacI transcriptional regulator (4) - - - 2.44 - 3.56 - -2.38 -  Krad1300 Transcriptional regulator, TetR family (1) - - 3.01 2.28 - - -3.88 -2.81 -  Krad2323 Regulatory protein LuxR (3) - - 2.39 - - - - - -2.56  Krad2529 Regulatory protein LacI (3) - - - 12.84 26.30 20.97 - -2.93 -2.28  Krad0304 Transcriptional regulator, DeoR family (1) - 2.44 6.89 -2.22 -4.25 - -2.37 -3.97 -4.61 |
