# Supplementary material for: Proteomic and Physiological Responses of Kineococcus radiotolerans to Copper
Source: PLoS One. 2010 Aug 26;5(8):e12427. doi: 10.1371/journal.pone.0012427 (PMC2928746; doi:10.1371/journal.pone.0012427)
Supplement: Table S6 — Median response of ribosomal proteins in K. radiotolerans during onset (16 hr) and mid (22 hr) exponential and stationary (32 hr) growth phases at varying concentrations of Cu(II). Response changes in protein abundance were calculated for all copper treatments relative to the no copper controls. The number of peptides detected for each protein is provided in parentheses. (0.04 MB DOC) [file pone.0012427.s006.doc]

**Table S6.** Median response of ribosomal proteins in *K. radiotolerans* during onset (16 hr) and mid (22 hr) exponential and stationary (32 hr) growth phases at varying concentrations of Cu(II). Response changes in protein abundance were calculated for all copper treatments relative to the no copper controls. The number of peptides detected for each protein is provided in parentheses.

| **16hr 22hr 32hr**  **Locus Protein** 0.1mM 0.75mM 1.5mM 0.1mM 0.75mM 1.5mM 0.1mM 0.75mM 1.5mM |
| --- |
| Krad0687 Ribosomal protein S10 (12) - 2.06 2.63 - - - 5.17 7.09 12.06  Krad0721 Ribosomal protein S9 (10) - - - -2.23 -2.27 - 7.83 7.83 4.64  Krad0663 Ribosomal protein L33 (3) - - - - - - 3.57 5.93 4.52  Krad1256 Ribosomal protein L31 (8) - - - - - - 3.26 3.17 5.63  Krad4513 Ribosomal protein L34 (1) - - - - - - - 2.80 4.28  Krad0697 Ribosomal protein S17 (11) - -2.36 - -3.44 -4.18 - 3.53 2.74 3.56  Krad1398 Ribosomal protein S16 (18) - - - - - - - 3.51 2.77  Krad3811 Sigma 54 modulation protein/ribosomal proteinS30EA (8) - - 2.68 - - 2.08 - - 5.09  Krad0700 Ribosomal protein L5 (16) - - - - - - - 2.08 3.08  Krad0694 Ribosomal protein S3 (35) - - - - - - - 2.68 2.24  Krad3421 Ribosomal protein S20 (4) - - - -14.02 - -2.81 5.17 4.61 -  Krad0705 Ribosomal protein S5 (23) - - - - - - 2.55 - 3.11  Krad1110 Ribosomal protein S4 (24) - - - - - - 2.30 - 2.29  Krad0698 Ribosomal protein L14 (12) - - - - - - 2.71 - 2.71  Krad4339 Ribosomal protein S6 (11) - - - - - - 2.37 - 2.75  Krad0682 Ribosomal protein S12 (8) - - - - - - 4.80 - 2.38  Krad1402 Ribosomal protein L19 (14) - - - - - - 2.15 - 2.25  Krad1465 Ribosomal protein S15 (9) - - - - - - 2.33 - 2.10  Krad0720 Ribosomal protein L13 (19) - - - - - - 2.15 - 2.06  Krad0678 Ribosomal protein L10 (24) - - - - - - - - 2.13  Krad0677 Ribosomal protein L1 (27) - - - - - - - - 2.17  Krad0285 Ribosomal protein S14 (2) - - - - - - 2.18 - 2.08  Krad0701 Ribosomal protein S14 (2) - - - - - - 2.18 - 2.08  Krad0714 Ribosomal protein S11 (14) - - - - - - 3.17 - 2.00  Krad0676 Ribosomal protein L11 (10) - - - - - - - - 2.07  Krad0679 Ribosomal protein L7/L12 (41) - - - - - - 2.55 - -  Krad0713 Ribosomal protein S13 (16) - - - - - - 2.08 - -  Krad0699 Ribosomal protein L24 (14) - - -2.72 - - - - - -  Krad1367 Ribosomal protein L28 (2) - - - - - - 2.74 - -  Krad1053 Ribosomal 5S rRNA E-loop binding proteinCtc/L25/TL5 (35) - - -2.02 - - - 2.20 - -  Krad0695 Ribosomal protein L16 (9) - - - - - - 2.20 - -  Krad0691 Ribosomal protein L2 (17) - - - - - - 2.02 - -  Krad0712 Ribosomal protein L36 (1) - - - - - - 2.00 - -  Krad0703 Ribosomal protein L6 (24) - - - - - - 2.47 - -  Krad0688 Ribosomal protein L3 (24) - - - - - - - -2.04 -  Krad3456 Ribosomal protein L27 (8) - - - - - - - -2.35 -  Krad0690 Ribosomal protein L25/L23 (10) - - - - - - - -2.48 -  Krad3168 Ribosomal protein L35 (5) - - - - - - 3.62 -2.78 -  Krad0693 Ribosomal protein L22 (16) - - - - - - 2.01 -3.66 -  Krad0702 Ribosomal protein S8 (17) - - - - - - - -3.34 -  Krad4337 Ribosomal protein S18 (7) - - - - -2.85 -2.69 2.86 -2.26 -2.03  Krad0683 Ribosomal protein S7 (20) - - - - - - 2.18 -3.81 -  Krad0692 Ribosomal protein S19 (16) - - -4.65 - -2.36 - 3.28 -4.33 -  Krad0696 Ribosomal protein L29 (18) - - - - - - - -4.65 -  Krad0689 Ribosomal protein L4/L1e (25) - - - - - - 2.10 -4.93 -  Krad0706 Ribosomal protein L30 (5) - - -3.32 - -3.76 - 2.23 -3.11 -3.18  Krad3457 Ribosomal protein L21 (12) - - - - - - 2.20 -13.48 -2.07  Krad1375 Ribosomal protein L32 (6) - - - - - - 2.30 -9.32 - |
