# Supplementary material for: Proteomic and Physiological Responses of Kineococcus radiotolerans to Copper
Source: PLoS One. 2010 Aug 26;5(8):e12427. doi: 10.1371/journal.pone.0012427 (PMC2928746; doi:10.1371/journal.pone.0012427)
Supplement: Table S7 — Median response of proteins involved in lipid metabolism in K. radiotolerans during onset (16 hr) and mid (22 hr) exponential and stationary (32 hr) growth phases at varying concentrations of Cu(II). Response changes in protein abundance were calculated for all copper treatments relative to the no copper controls. The number of peptides detected for each protein is provided in parentheses. (0.04 MB DOC) [file pone.0012427.s007.doc]

**Table S7.** Median response of proteins involved in lipid metabolism in *K. radiotolerans* during onset (16 hr) and mid (22 hr) exponential and stationary (32 hr) growth phases at varying concentrations of Cu(II). Response changes in protein abundance were calculated for all copper treatments relative to the no copper controls. The number of peptides detected for each protein is provided in parentheses.

| **16hr 22hr 32hr**  **Locus Protein** 0.1mM 0.75mM 1.5mM 0.1mM 0.75mM 1.5mM 0.1mM 0.75mM 1.5mM |
| --- |
| Krad0139 Short-chain dehydrogenase/reductase SDR(8) - - 2.06 - - - - - 4.94  Krad2814 Short-chain dehydrogenase/reductase SDR (1) 2.38 - - - - - - - 2.26  Krad1386 Rhamnulose-1-phosphate aldolase/alcoholdehydrogenase (3) - - 4.08 2.43 - 2.36 2.20 -13.48 -2.07  Krad2087 Short-chain dehydrogenase/reductase SDR (4) - - - - - - -2.85 - -  Krad4028 Acetyl-CoA acetyltransferase (5) - - - - - - - 2.24 2.22  Krad1046 4-diphosphocytidyl-2C-methyl-D-erythritolkinase (1) - - - - - - -2.47 - -  Krad1123 Hydroxymethylbutenyl pyrophosphate reductase (6) - - - - - - - 2.96 3.05  Krad1429 1-hydroxy-2-methyl-2-(E)-butenyl 4-diphosphatesynthase (27) - - - - - - - 2.63 3.13  Krad1578 Deoxyxylulose-5-phosphate synthase (15) - 2.05 - - - - - - 2.91  Krad3227 Polyprenyl synthetase (17) - - 2.29 - - 2.97 3.26 3.17 5.63  Krad0262 Short-chain dehydrogenase/reductase SDR (14) - - 2.05 - - - - - -  Krad0561 Transferase hexapeptide repeat containing protein (9) - - 2.01 - 2.11 3.93 6.24 7.71 5.64  Krad0744 Short-chain dehydrogenase/reductase SDR (14) - - - - - - - - 2.27  Krad0797 Short-chain dehydrogenase/reductase SDR (8) - - - - - 2.35 2.18 -3.81 -  Krad0828 Short-chain dehydrogenase/reductase SDR (2) - 24.45 - -2.07 -2.28 - -3.04 - -  Krad1454 Short-chain dehydrogenase/reductase SDR (8) - - - - - - -2.25 - -  Krad1634 Short-chain dehydrogenase/reductase SDR (2) - - - - 2.35 2.19 - 2.42 3.96  Krad1682 3-Oxoacyl-synthaseIII domain protein (6) - - - - - - -2.73 - -3.44  Krad2779 Short-chain dehydrogenase/reductase SDR (1) 11.49 66.82 241.70 134.38 33.75 78.35 56.75 150.80 73.40  Krad3162 Short-chain dehydrogenase/reductase SDR (9) - - - - 3.04 - - 3.46 3.56  Krad3254 Short-chain dehydrogenase/reductase SDR (3) - - - - - - - - 3.14  Krad3343 Acyl transferase (15) - - - - - - - -2.37 -2.31  Krad3344 3-oxoacyl-(acyl-carrier-protein) synthase III (13) - - - - - - - - 2.79  Krad3346 Beta-ketoacyl synthase (26) - - 3.58 2.28 - 2.37 2.20 7.66 6.53  Krad3641 Short-chain dehydrogenase/reductase SDR (9) - - -2.79 - - - -2.32 - -  Krad4023 Short-chain dehydrogenase/reductase SDR (3) - - 2.05 - - - - - -  Krad4324 Short-chain dehydrogenase/reductase SDR (5) - - - - - - - 3.84 3.68  Krad4417 Short-chain dehydrogenase/reductase SDR (1) -2.07 - - - 2.05 - - 2.29 2.76  Krad3502 Acetyl-CoA acetyltransferase (8) - 3.82 5.69 2.92 4.21 - - - -  Krad3503 3-hydroxyacyl-CoA dehydrogenase NAD-binding (15) - - - - - - - 4.23 4.84  Krad3505 Acyl-CoA dehydrogenase domain protein (13) - - - - - - 2.53 - -  Krad0345 Alcohol dehydrogenase GroES domain protein (4) - - - - - 2.30 2.14 - 3.49  Krad0730 Alpha-beta hydrolase fold (2) - - - - 3.05 - - 3.03 5.04  Krad1008 Glycerol kinase (28) - - 2.15 - - - - 2.21 2.36  Krad1009 FAD dependent oxidoreductase (35) - - - - - - - 2.11 2.60  Krad1150 O-methyltransferase family 3 (7) - 2.97 - - - 2.20 - 3.79 4.16  Krad1916 3-hydroxyacyl-CoA dehydrogenase NAD-binding (35) - - - - - - - - 3.09  Krad1919 Glycerone kinase (23) - - - - - - -3.50 - -  Krad2223 Alpha-beta hydrolase fold (1) - - -3.48 - - - - -  Krad2708 2,5-didehydrogluconate reductase (2) -2.34 - - - 4.66 - - - 5.09  Krad2782 Thioesterase superfamily protein (1) - - -2.03 - - - - 2.05 2.11  Krad3116 Phospholipid/glycerol acyltransferase (8) - - - - - - - - 4.35  Krad3235 Phospholipid/glycerol acyltransferase (3) - - 4.24 2.46 - 3.35 4.02 2.84 -  Krad3540 Palmitoyl-CoA hydrolase (5) - - -3.60 - -2.17 - -3.04 - -  Krad4000 Glycerone kinase (11) - 2.12 4.00 2.43 - 2.02 - - -  Krad4349 2,5-didehydrogluconate reductase (17) - - 2.25 - - - - - 3.24 |
